# Development and validation of an infectious disease control competency scale for public health professionals

**DOI:** 10.1186/s41256-024-00381-y

**Published:** 2024-09-26

**Authors:** Yiguo Zhou, Wan-Xue Zhang, Shan-Shan Zhang, Ning-Hua Huang, Jing Zeng, Han Yang, Qin-Yi Ma, Le Ao, Ya-Qiong Liu, Juan Du, Xiao-Ling Tian, Qing-Bin Lu, Fuqiang Cui

**Affiliations:** 1 Department of Health Policy and Management, School of Public Health, Peking University, Beijing, China; 2 Department of Epidemiology and Biostatistics, School of Public Health, Peking University, Beijing, China; 3 Centre for Infectious Disease and Policy Research and Global Health and Infectious Diseases Group, Peking University, Beijing, China; 4 Department of Laboratorial Science and Technology and Vaccine Research Centre, School of Public Health, Peking University, No. 38 Xue-Yuan Road, Haidian District, Beijing, 100191 China; 5 Institute for Immunization Program, Inner Mongolia Centre for Disease Control and Prevention (The Academy of Preventive Medicine Sciences In Inner Mongolia), South Yongping Road, Xincheng District, Hohhot, 010080 China; 6 Key Laboratory of Epidemiology of Major Diseases (Peking University), Ministry of Education, Beijing, China

**Keywords:** Public health professionals, Infectious diseases, Competency, Delphi, Exploratory factor analysis, Confirmatory factor analysis

## Abstract

**Background:**

Infectious diseases persistently pose global threats, and it is imperative to accelerate the professionalization of public health workforce. This study aimed to develop and validate the infectious disease control competency scale (IDCCS) for public health professionals to fill a theoretical gap and elevate practical capabilities by informing public health professionals’ development goals.

**Methods:**

The initial item pool was generated through a literature review, and categorized into three dimensions (knowledge, practical skills, and leadership) based on the competency iceberg model and public health leadership framework. A two-round Delphi process was conducted to determine indicators within the scale. A pilot survey was utilized for item analysis and exploratory factor analysis (EFA). A formal survey was employed for confirmatory factor analysis (CFA). The weight value of each indicator was calculated using the analytic hierarchy process.

**Results:**

An initial scale with three primary items, 14 secondary items, and 81 tertiary items was generated. Twenty experts participated in the two rounds of the Delphi process. Authority coefficients exceeded 0.9 in both rounds. Kendall's *W* was 0.29 and 0.19, respectively (both *P* < 0.001). Item analysis presented a Cronbach's Alpha of 0.98, with corrected item-total correlation coefficients ranging from 0.33 to 0.78. EFA demonstrated that cumulative variance explanations for the four primary dimensions (knowledge, practical skills, leadership, and personal quality) were 77.463%, 73.976%, 81.174%, and 68.654%, respectively. CFA indicated that all composite reliability values and average variance extracted surpassed 0.8 and 0.5, respectively. The standardized factor loadings of the items ranged from 0.630 to 0.977. Among the seven model fit indices, each of the four dimensions satisfied at least five criteria. A final three-level scale comprising four primary items, 14 secondary items, and 64 tertiary items was constructed. The weight values for the four primary items were 0.4064, 0.2878, 0.2082, and 0.0981, respectively.

**Conclusions:**

The IDCCS was established to evaluate the competencies of knowledge, practical skills, leadership, and personal quality for public health professionals in infectious disease control. This scale demonstrates good reliability and validity, and can be used for performance evaluation, recruitment processes, curriculum development, and individual self-assessment.

**Supplementary Information:**

The online version contains supplementary material available at 10.1186/s41256-024-00381-y.

## Introduction

Infectious diseases persistently pose global threats, manifested in periodic outbreaks of epidemics such as influenza and coronavirus disease 2019, and compounded by rising antimicrobial resistance [1]. Despite biomedical advances reducing associated morbidity and mortality, newly emerging and re-emerging infectious diseases with pandemic potential remain a pressing public health concern in our interconnected world [2]. Enhancing outbreak prediction and response requires more advanced skills among public health professionals, including utilizing new technologies such as internet-based surveillance systems and computational modeling to analyze pathogen transmission and impacts [3].

Currently, numerous countries are experiencing significant shortages in their public health workforce, both in terms of quantity and quality, which is hindering efforts to control infectious diseases. Data reveal over a 15% decline in the public health workforce in the United States alone over the past decade [4]. The World Health Organization (WHO) estimated a global shortage of 18 million health workers by 2030 [5]. Moreover, inadequate professional training is widespread among practitioners. Nearly two-thirds of personnel who carry out fundamental public health functions in some countries lack formal specialized public health education [6]. With intensifying structural challenges such as population aging and emerging infectious diseases, it is imperative to enhance capabilities in these fields through improved health workforce professionalization.

After outbreak of severe acute respiratory syndrome in 2003, China has been investing in infectious disease control, exemplified by revising the Law on the Prevention and Treatment of Infectious Diseases, enhancing emergency response systems, and establishing national disease reporting and surveillance networks [7, 8]. However, deficiencies persist regarding capacities for early outbreak detection and response, technical expertise, and interagency coordination [9]. Additionally, public health agencies in China also suffer from workforce attrition and skill gaps. Even model public health systems, such as that of Beijing, struggle to recruit and retain high-caliber personnel [10]. Consequently, the State Council emphasized key initiatives, including establishing high-caliber public health talent cultivation projects and training core experts from Centress for Disease Control and Prevention (CDCs) in 2023 guidelines on "Promoting High-Quality Development of Disease Prevention and Control" [11].

Several international competency frameworks for public health personnel exist, including the WHO's guidelines and the United Kingdom's Public Health Skills and Knowledge Framework [12, 13]. However, these frameworks focus on generalist skills without addressing specialized technical capacities such as infectious disease control. There was minimal research explicitly examining outbreak response competencies for public health professionals in China, contrasting with substantial assessments among doctors and nurses. More than half of these existing assessments relied on non-standardized tools lacking rigorous validation [14–17]. As jurisdictions such as Shanghai start planning specialized assessments among disease control experts, public health emergency responders and other critical personnel [18], a pressing need arises for unified, rigorously-developed instruments tailored to infectious disease competencies, which also capture the leadership capabilities central to guiding multisectoral response efforts. The absence of definitive standards underscores the significance of developing a competency scale for infectious disease control. This scale would fill a theoretical gap and enhance practical capabilities by informing public health professionals’ development goals. Crucially, in the face of changing public health threats, a validated tool is essential for strengthening overall preparedness to infectious diseases.

In this study, we aimed to develop a competency scale focusing on infectious disease prevention and control among public health professionals based on the competency iceberg model [19] and the frame of public health leadership [20]. Public health professionals engaged in infectious disease control were enrolled for scale validation.

## Methods

### Study design

**Fig. 1:**
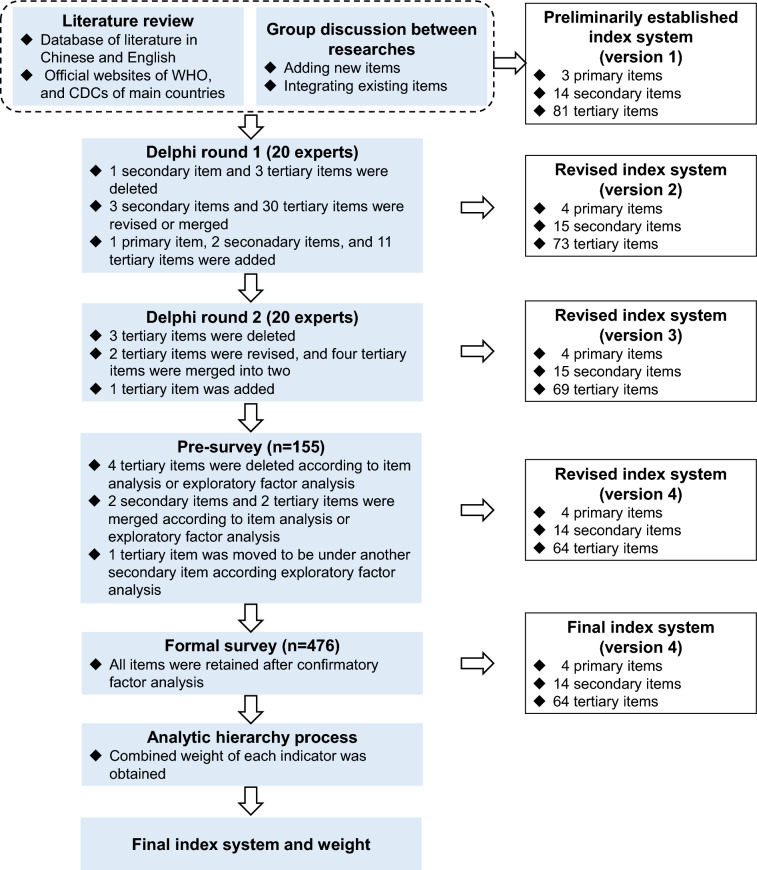
The study flow chart

A research group was assembled to construct and revise the infectious disease control competency scale (IDCCS), comprising two experts specializing in infectious disease prevention and control, two PhD students, and three master's students. This study adhered to published recommendations for scale development and reporting [21, 22]. The study encompassed four phases (Fig. 1): construction of the initial scale, Delphi process [23], evaluation and validation of the scale, and the analytic hierarchy process (AHP) [24].

### Construction of the initial scale

Relevant initial items were gathered from the expert experience, literature [20, 25–28] and documents [29] related to personnel competencies in infectious disease prevention and control or public health emergency management. The sources included PubMed, China National Knowledge Infrastructure, Google Scholar, and official websites of CDCs in the United States, European Union, and China. Based on the competency iceberg model [19] and the leadership attributes of public health professionals, an initial scale with a three-level structure was developed through research group discussion.

### Delphi process

Two rounds of Delphi process were conducted via email between March and May 2023. Experts meeting the following criteria were invited: (1) possessing over 10 years of work or research experience in the prevention and control of infectious disease; (2) holding senior titles, such as associate professor, professor, or equivalent senior positions in research or healthcare institutions. Experts' regional distribution, professional background, and multidisciplinary expertise were considered for representativeness and authority. If an expert who completed the first round could not participate in the second round, an alternative expert with an equivalent or stronger professional background was identified. A 5-point Likert scale [30] measured the importance of each item, and the suggestions of experts were collected. Experts reported judgment criteria (Ca) and familiarity (Cs), with the authority coefficient (Cr) calculated as (Ca + Cs)/2 (Cr ≥ 0.7 considered acceptable). After each round, items were adjusted based on the critical value method and the suggestions of experts, retaining items with average importance score ≥ 4.05, coefficient of variation (CV) ≤ 0.17, and full mark rate ≥ 26.0% in the first round; and average score ≥ 4.34, CV ≤ 0.14, and full mark rate ≥ 41.9% in the second round. If any one of these three indicators was unsatisfied, the research group discussed adjustment methods. The degree of coordination of experts’ scores was determined by Kendall’s coefficients of concordance (Kendall’s *W*).

### Evaluation and validation of the scale

In June 2023, a pilot survey was conducted in Guizhou and Inner Mongolia, China. The subjects in the pilot study were individuals aged 35–50 years without disabilities or severe illnesses. They were involved in public health emergency or infectious disease control work at provincial or municipal CDCs and held intermediate or higher professional titles. All subjects provided informed consent. An electronic questionnaire was developed on the Wen-Juan Xing platform (Changsha Ranxing Information Technology Co., Ltd., Hunan, China) and distributed through internal CDC communication channels (e.g., WeChat group). The questionnaire included sociodemographic characteristics and the revised scale version after the Delphi method.

For exploratory factor analysis (EFA), a sample size of 150–200 is generally considered sufficient when the data set exhibits high communalities (above 0.5) or when there is a ratio of approximately 10 subjects per item with factor loadings of |0.4| or higher [21]. Data were analyzed using SPSS 23.0 (IBM Corporation, New York, NY, United States) and STATA 17.0 (StataCorp, TX, United States). Item analysis and EFA were used to refine and improve the scale structure. Specifically, *t*-tests were used to compare the upper 27% and lower 27% score groups. Corrected item-total correlation coefficients between individual items and the overall scale score were calculated, with coefficients above 0.4 indicating satisfactory results. The internal consistency of items was assessed by Cronbach's Alpha coefficient, where values above 0.7 indicated strong internal consistency. Cronbach's Alpha was calculated after removing individual items, and higher coefficients might indicate items that could be removed. The Kaiser–Meyer–Olkin (KMO) test and Bartlett's test of sphericity (*P* < 0.05) were used to assess EFA adequacy, with KMO values > 0.7 indicating suitability for factor analysis. A fixed number of factors were extracted according to the secondary item count in each dimension. Principal component analysis (PCA) with varimax orthogonal rotation was employed for data extraction. Items with factor loadings < 0.45 required adjustment. Items failing item analysis and EFA underwent research team discussion to determine adjustment methods. EFA was rerun after adjustment until all items fitted the preset factor frame.

The formal survey in July 2023 across 11 provinces targeted the same population. A minimum of five cases per item was recommended for sample size [21]. The questionnaire included the updated scale version after item analysis and EFA.

Amos 28 software (IBM Corporation, New York, NY, United States) was used to conduct the confirmatory factor analysis (CFA). Convergent validity was assessed through standardized factor loadings, composite reliability (CR), and average variance extracted (AVE). Satisfactory discriminant validity was determined by a higher square root of AVE than the correlation coefficients between factors. Convergent validity criteria included CR values ≥ 0.70 and AVE values ≥ 0.50. CFA model fit appropriateness was determined by: Chi-square to degree of freedom ratio (χ^2^/df) < 3, goodness of fit index (GFI) > 0.9, comparative fit index (CFI) > 0.9, normed fit index (NFI) > 0.9, Tucker-Lewis index (TLI) > 0.9, standardized root mean square residual (SRMR) < 0.05, and root mean square error of approximation (RMSEA) < 0.08. Meeting most of these indicators signified acceptable model fit.

### Analytic hierarchy process

The analytic hierarchy process (AHP) was used to define the weight of each indicator [31]. For this three-level structure, all indicators were compared pairwise within each level according to the mean of expert scoring in the second Delphi round. Saaty's fundamental 9-point scale was adopted to determine the relative importance of one indicator compared to another [32]. Δ represents the difference in mean importance scores between any two indicators. The scoring standards were as follows: a score of 3 was assigned when 0.25 < Δ ≤ 0.50; 5 when 0.75 < Δ ≤ 1.00; 7 when 1.25 < Δ ≤ 1.50; and 9 when Δ > 1.75. For Δ values falling between these ranges, the scores were interpolated as 2, 4, 6, or 8, accordingly. The established expert judgment matrixes were entered into yaahp 10.1 (yaahp software, Taiyuan, Shanxi, China) to calculate the consistency ratio of each judgment matrix and the combined weight of each indicator.

## Results

### Summaries of the initial scale

The research group categorized the initial index pool into three dimensions: knowledge, practical skills, and leadership. The preliminary index pool comprised three primary items, 14 secondary items, and 81 tertiary items (Supplementary Table 1).

### Scale modification via the Delphi method

#### Delphi round one

Twenty experts participated in the first round from 24 experts we initially contacted, yielding a response rate of 83.3%. The majority were male (13/20, 65%), aged over 50 years (14/20, 70%), working in CDCs or research institutions (19/20, 95%), and having a background in public health and preventive medicine (18/20, 90%). 95%(19/20) of the experts held a master's degree or above, 90% (18/20) possessed senior professional titles, and 85% (17/20) had at least 20 years of work experience (Table 1). The average Cr values for the three primary dimensions were 0.935, 0.933, and 0.908, respectively (Supplementary Table 2), representing a high authority.

**Table 1:** Characteristics of Delphi experts

Characteristics	Round 1		Round 2	
	Frequency, n	Percentage, %	Frequency, n	Percentage, %
Gender				
Male	13	65.0	15	75.0
Female	7	35.0	5	25.0
Age, years				
30–39	2	10.0	1	5.0
40–49	4	20.0	5	25.0
50–59	8	40.0	12	60.0
≥ 60	6	30.0	2	10.0
Institution type				
CDC	10	50.0	11	55.0
Maternal and Child Healthcare institution	0	0	1	5.0
Colleges, universities, or research institutions	9	45.0	8	40.0
Others	1	5.0	0	0
Major type				
Public health and preventive medicine	18	90.0	18	90.0
Clinical medicine	1	5.0	1	5.0
Pathogenic microorganisms and other medical disciplines	0	0	1	5.0
Social medicine and healthcare management	1	5.0	0	0.0
Education level				
Doctorate	9	45.0	13	65.0
Master's degree	10	50.0	4	20.0
Bachelor's degree	1	5.0	3	15.0
Professional title				
Senior	18	90.0	19	95.0
Sub-senior	2	10.0	1	5.0
Years of work				
< 10	0	0	0	0.0
10–19	3	15.0	2	10.0
20–29	7	35.0	8	40.0
≥ 30	10	50.0	10	50.0
Familiarity with infectious disease prevention and control or health emergency response				
Highest degree	14	70.0	15	75.0
High degree	6	30.0	5	25.0

Sub-senior professional title: experienced professionals who have made significant contributions to their discipline and exhibited leadership capabilities (ranks 7–5); senior title: the highest level, reserved for exceptional professionals with distinguished accomplishments, recognized expertise, and substantial leadership roles within their organizations or fields (ranks 4–1)

*CDC* Centre for Disease Control and Prevention

Across all indicators, experts’ mean importance scores ranged from 3.32 to 4.95, with CV values ranged from 0.04 to 0.24, and full mark proportions ranging from 0 to 95% (Table 2). According to the critical value method and experts’ suggestions, one secondary item and three tertiary items were deleted, three secondary items and 30 tertiary items were revised or merged, and one primary item, two secondary items, and 11 tertiary items were newly added (Supplementary Table 3). A new primary item named "Personal Quality" was introduced, encompassing two secondary items (professional qualifications and professional quality). Kendall’s *W* was 0.285 (*P* < 0.001), indicating an acceptable coordination among experts (Supplementary Table 4). The revised scale after the first Delphi round comprised four primary items, 15 secondary items, and 73 tertiary items (Supplementary Table 5).

**Table 2:** Experts’ scores in the first round of Delphi

Primary items	Items	Significance				
		Median	Mean	SD	CV	Consensus (%score of 5)
A Knowledge	A Knowledge	5	4.85	0.36	0.07	85
B Practical skills	B Practical skills	5	4.80	0.40	0.08	80
C Leadership	C Leadership	5	4.65	0.48	0.10	65
A Knowledge	A1 Basic knowledge of infectious diseases	5	4.75	0.54	0.11	80
	A2 Basic knowledge of public health emergency management	5	4.60	0.49	0.11	60
	A3 National plan and systems for public health emergencies in China (emergency plan, emergency management system, operation mechanism and legal system)	4	4.25	0.54	0.13	30
B Practical skills	B1 Infectious diseases prevention and emergency preparedness	5	4.65	0.48	0.10	65
	B2 Infectious diseases surveillance and early warning	5	4.85	0.36	0.07	85
	B3 Public health response to infectious diseases	5	4.70	0.46	0.10	70
	B4 Scientific research ability	4	3.95	0.67	0.17	20
	B5 Business guidance ability	5	4.45	0.67	0.15	55
C Leadership	C1 Leadership fundamentals	5	4.45	0.67	0.15	55
	C2 Decision-making ability	5	4.45	0.74	0.17	55
	C3 Team mobilization ability	5	4.50	0.74	0.16	60
	C4 Communication skills	5	4.60	0.58	0.13	65
	C5 Self-regulation and interpersonal coordination abilities	5	4.50	0.59	0.13	55
	C6 Team learning and development	5	4.50	0.59	0.13	55
A Knowledge	A1_1 Pathogenic biology of common infectious disease pathogens	5	4.65	0.57	0.12	70
	A1_2 Criteria for judging infectious source of infectious diseases	5	4.80	0.51	0.11	85
	A1_3 Criteria for judging transmission route of pathogens	5	4.85	0.36	0.07	85
	A1_4 Susceptible populations for common infectious diseases	5	4.75	0.43	0.09	75
	A1_5 Criteria for judging aggregates epidemics and outbreaks of infectious diseases	5	4.70	0.46	0.10	70
	A1_6 Influencing factors of epidemic spread of infectious diseases	5	4.85	0.36	0.07	85
	A1_7 Common prevention and control measures for infectious diseases	5	4.80	0.51	0.11	85
	A1_8 Clinical manifestations of common infectious diseases	5	4.40	0.73	0.17	55
	A1_9 Diagnostic criteria and differential diagnosis of common infectious diseases	4	4.25	0.62	0.15	35
	A1_10 Treatment principles of common infectious diseases	4	3.85	0.65	0.17	15
	A2_1 Fundamentals of public health emergency management	5	4.65	0.57	0.12	70
	A2_2 Classification of infectious diseases surveillance	4	4.40	0.49	0.11	40
	A2_3 Steps of early warning of public health emergency	4	4.35	0.48	0.11	35
	A2_4 Theories of health emergency management	4	4.15	0.65	0.16	30
	A2_5 Theories of crisis decision making	4	4.25	0.70	0.16	40
	A2_6 Theories of risk assessment	4	4.35	0.65	0.15	45
	A2_7 Theories of risk communication	5	4.70	0.56	0.12	75
	A2_8 Command, coordination and control of health emergency	5	4.70	0.46	0.10	70
	A3_1 Responsibilities of disease control personnel in National Emergency Response Plan for Public Health Emergencies	4.5	4.35	0.73	0.17	50
	A3_2 Responsibilities of disease control personnel in National Medical Rescue Scheme for Public Health Emergencies	4	3.95	0.74	0.19	25
	A3_3 Understanding of responsibilities of disease control personnel in Law on Prevention and Control of Infectious Diseases of the People's Republic of China	4	4.15	0.73	0.18	35
	A3_4 Responsibilities of disease control personnel in Regulations on Emergency Response to Public Health Emergencies	4	4.25	0.62	0.15	35
	A3_5 Prevention and preparedness mechanism for public health emergencies in China	4	4.35	0.65	0.15	45
	A3_6 Surveillance and early warning mechanism for public health emergencies in China	4.5	4.40	0.66	0.15	50
	A3_7 Response and rescue mechanism for public health emergencies in China	4	4.15	0.65	0.16	30
	A3_8 Aftermath assessment mechanism for public health emergencies in China	4	3.95	0.74	0.19	25
	A3_9 Health emergency system in China	4	4.20	0.51	0.12	25
B Practical skills	B1_1 Development of emergency plans	4	4.35	0.65	0.15	45
	B1_2 Health promotion	4	4.00	0.77	0.19	30
	B1_3 Receiving professional training	4	4.45	0.50	0.11	45
	B1_4 Participating in emergency drills	5	4.45	0.67	0.15	55
	B1_5 Emergency capacity assessment	4.5	4.45	0.59	0.13	50
	B2_1 Clarifying the content and process of infectious diseases surveillance	5	4.80	0.40	0.08	80
	B2_2 Ability to detect abnormal signals of public health emergencies	5	4.95	0.22	0.04	95
	B2_3 Clarifying the reporting process for public health emergencies	4	4.30	0.56	0.13	35
	B2_4 Determining reliability of information sources for emerging infectious diseases	4	4.25	0.83	0.20	45
	B2_5 Ability to extract key information from selected information sources	5	4.80	0.40	0.08	80
	B2_6 Predicting occurrence and epidemic trends of infectious diseases based on surveillance information	5	4.70	0.56	0.12	75
	B3_1 Mastering knowledge and skills of personal protection	5	4.65	0.57	0.12	70
	B3_2 Mastering principles of setting up isolation wards for infectious diseases	5	4.60	0.49	0.11	60
	B3_3 Ability to properly handle items or corpses involving infectious pathogens	4	4.20	0.68	0.16	35
	B3_4 Mastering methods of environmental disinfection and sampling	4	4.30	0.64	0.15	40
	B3_5 Correctly implementing isolation measures for various infectious disease patients	5	4.55	0.50	0.11	55
	B3_6 Ability to correctly carry out epidemiological investigations and write investigation reports	5	4.65	0.48	0.10	65
	B3_7 Analyzing the situation of public health incidents and proposing targeted prevention and control measures	5	4.80	0.40	0.08	80
	B3_8 Clarifying on-site processing procedures for public health emergencies	5	4.60	0.58	0.13	65
	B4_1 Having honorary titles of expert	3	3.32	0.65	0.20	0
	B4_2 Undertaking research projects	4	4.00	0.71	0.18	20
	B4_3 Publishing research papers	4	4.05	0.67	0.17	25
	B4_4 Authoring professional publications	4	3.60	0.80	0.22	10
	B4_5 Obtaining national patents	3	3.40	0.80	0.24	5
	B4_6 Winning research awards	3.5	3.50	0.67	0.19	5
	B4_7 Research design ability	5	4.60	0.58	0.13	65
	B4_8 Chinese paper writing ability	4	4.10	0.77	0.19	30
	B4_9 English paper writing ability	4	3.80	0.60	0.16	10
	B4_10 Understanding domestic and foreign status and trends in the profession	5	4.70	0.56	0.12	75
	B5_1 Experience in guiding students	4	3.70	0.71	0.19	10
	B5_2 Experience in guiding subordinate in training and learning	4	4.00	0.55	0.14	15
	B5_3 Training guidance ability	4.5	4.40	0.66	0.15	50
C Leadership	C1_1 Task assignment with division of responsibilities	4	4.35	0.65	0.15	45
	C1_2 Ability to obtain resources needed for teamwork	5	4.45	0.67	0.15	55
	C1_3 Ability to allocate and dispatch resources needed for teamwork	5	4.55	0.50	0.11	55
	C1_4 Supervising and adjusting project implementation	4.5	4.45	0.59	0.13	50
	C2_1 Systematic understanding of current public health issues	5	4.55	0.50	0.11	55
	C2_2 Integrating different perspectives during decision making	5	4.55	0.74	0.16	70
	C2_3 Clarifying problems to be solved and expected outcomes of decisions	4.5	4.45	0.59	0.13	50
	C2_4 Ability to formulate alternative plans and select the optimal one	5	4.65	0.48	0.10	65
	C2_5 Ability to execute project plans	5	4.35	0.85	0.20	55
	C3_1 Creating an environment conducive to opinion exchange within the department	4	4.20	0.60	0.14	30
	C3_2 Having characteristics to enable effective team leadership like integrity, enthusiasm, honesty, caring, trustworthiness, sense of responsibility, etc	5	4.45	0.67	0.15	55
	C3_3 Establishing effective team motivation models including listening, dialoguing, negotiating, rewarding, encouragement, inspiration, etc	5	4.50	0.59	0.13	55
	C4_1 Demonstrating excellent writing, communication and presentation skills	4	4.15	0.73	0.18	35
	C4_2 Regularly communicating information regarding public health needs, goals, achievements and major crises to target audience through media	4.5	4.40	0.66	0.15	50
	C4_3 Effectively applying negotiation skills in resolving disputes	4.5	4.40	0.66	0.15	50
	C5_1 Understanding the impact of own behaviors or reactions on team members	4	4.20	0.68	0.16	35
	C5_2 Giving proper feedback to criticisms on own behaviors or performance by others	4	4.20	0.68	0.16	35
	C5_3 Demonstrating adaptability and ability to motivate myself when facing threats or pressure	4.5	4.30	0.84	0.20	50
	C6_1 Identifying opportunities for team growth, innovation, reform and development	4	4.35	0.65	0.15	45
	C6_2 Creating opportunities for teams to learn and improve together	4	4.30	0.64	0.15	40
	C6_3 Helping members clarify thinking and turn ideas into feasible plans	4.5	4.45	0.59	0.13	50
	C6_4 Post-incident learning and summarization abilities	5	4.70	0.46	0.10	70

*SD* standard deviation, *CV* coefficient of variation

#### Delphi round two

Twenty experts participated in the second round from 27 experts contacted, resulting in a response rate of 74.1%, with similar basic characteristics to those in the first round (Table 1). The average Cr values of four primary dimensions were 0.938, 0.925, 0.933, and 0.945, respectively (Supplementary Table 6), representing a high authority.

Across all indicators, experts’ mean importance scores ranged from 3.85 to 4.85, with CV values ranged from 0.07 to 0.18, and full mark proportions ranging from 10 to 85% (Table 3). Based on the critical value method and experts’ suggestions, three tertiary items were deleted, two tertiary items were revised, four tertiary items were merged into two, and one tertiary item was newly added (Supplementary Table 7). Kendall’s *W* was 0.192 (*P* < 0.001), indicating an acceptable coordination among experts (Supplementary Table 8). The revised scale after the second Delphi round comprised four primary items, 15 secondary items, and 69 tertiary items (Supplementary Table 9).

**Table 3:** Experts’ scores in the second round of Delphi

Primary items	Items	Significance				
		Median	Mean	SD	CV	Consensus (%score of 5)
A Knowledge	A Knowledge	5	4.85	0.36	0.07	85
B Practical skills	B Practical skills	5	4.80	0.40	0.08	80
C Leadership	C Leadership	5	4.70	0.56	0.12	75
D Personal quality	D Personal quality	4.5	4.45	0.59	0.13	50
A Knowledge	A1 Knowledge of infectious diseases	5	4.75	0.43	0.09	75
	A2 Knowledge of public health emergency management	5	4.65	0.48	0.10	65
	A3 Laws, plans and mechanisms for responding to public health emergencies	5	4.60	0.49	0.11	60
B Practical skills	B1 Infectious diseases prevention and emergency preparedness	5	4.75	0.43	0.09	75
	B2 Infectious diseases surveillance and early warning	5	4.80	0.40	0.08	80
	B3 Public health response to infectious diseases	5	4.85	0.36	0.07	85
	B4 Scientific research ability	4	4.15	0.65	0.16	30
	C1 Leadership fundamentals	5	4.75	0.43	0.09	75
C Leadership	C2 Decision-making ability	5	4.75	0.54	0.11	80
	C3 Team mobilization ability	5	4.70	0.46	0.10	70
	C4 Communication skills	5	4.85	0.36	0.07	85
	C5 Self-regulation and interpersonal coordination abilities	5	4.55	0.50	0.11	55
	C6 Team learning and development	5	4.55	0.50	0.11	55
D Personal quality	D1 Professional qualifications	4	4.00	0.55	0.14	15
	D2 Professional quality	5	4.55	0.50	0.11	55
A Knowledge	A1_1 Basic knowledge of infectious diseases (etiology knowledge, judgment of infection source and transmission route, understanding of susceptible population, etc.)	5	4.85	0.36	0.07	85
	A1_2 Criteria for judging aggregates epidemics and outbreaks of infectious diseases (including nosocomial infections)	5	4.70	0.56	0.12	75
	A1_3 Influencing factors of epidemic spread of infectious diseases	5	4.65	0.57	0.12	70
	A1_4 Common prevention and control measures for infectious diseases	5	4.75	0.54	0.11	80
	A1_5 Clinical manifestations of common infectious diseases	5	4.40	0.73	0.17	55
	A1_6 Diagnostic criteria and differential diagnosis of common infectious diseases	4	4.20	0.75	0.18	40
	A2_1 Fundamentals of public health emergency management	5	4.70	0.46	0.10	70
	A2_2 Different ways of infectious disease surveillance	4	4.45	0.50	0.11	45
	A2_3 Steps of early warning of public health emergency	5	4.45	0.67	0.15	55
	A2_4 Theories of health emergency management	4	4.30	0.56	0.13	35
	A2_5 Theories of crisis decision making	4	4.40	0.58	0.13	45
	A2_6 Theories of risk assessment	5	4.55	0.67	0.15	65
	A2_7 Theories of risk communication	5	4.75	0.54	0.11	80
	A2_8 Command, coordination and control of health emergency	5	4.80	0.40	0.08	80
	A3_1 The knowledge of relevant laws and regulations on infectious disease prevention and control and health emergency in China	5	4.55	0.50	0.11	55
	A3_2 The knowledge of the prevention and control of infectious diseases and related health emergency plans in China	4.5	4.50	0.50	0.11	50
	A3_3 Prevention and preparedness mechanism for public health emergencies in China	4	4.35	0.57	0.13	40
	A3_4 Surveillance and early warning mechanism for public health emergencies in China	5	4.50	0.59	0.13	55
	A3_5 Response and rescue mechanism for public health emergencies in China	4	4.30	0.64	0.15	40
B Practical skills	B1_1 Development of emergency plans	4	4.40	0.49	0.11	40
	B1_2 Health popularization on infectious diseases and public health emergencies	4	4.35	0.65	0.15	45
	B1_3 Receiving professional training	5	4.65	0.48	0.10	65
	B1_4 Participating in emergency drills	5	4.55	0.67	0.15	65
	B1_5 Emergency capacity assessment	5	4.55	0.50	0.11	55
	B2_1 Clarifying the content and process of infectious diseases surveillance	5	4.80	0.40	0.08	80
	B2_2 Ability to detect abnormal signals of public health emergencies	5	4.80	0.40	0.08	80
	B2_3 Clarifying the reporting process for public health emergencies	5	4.55	0.67	0.15	65
	B2_4 Determining the reliability of information sources on infectious diseases	5	4.65	0.65	0.14	75
	B2_5 Ability to extract key information from selected information sources	5	4.80	0.51	0.11	85
	B2_6 Predicting occurrence and epidemic trends of infectious diseases based on surveillance information	5	4.75	0.43	0.09	75
	B3_1 Mastering knowledge and skills of personal protection	5	4.70	0.46	0.10	70
	B3_2 Clarifying on-site processing procedures for public health emergencies	5	4.80	0.40	0.08	80
	B3_3 Mastering the principles of defining epidemic areas	5	4.80	0.40	0.08	80
	B3_4 Familiar with the methods of case management and disinfection in the field environment	5	4.55	0.74	0.16	70
	B3_5 Correctly implementing the collection, transportation and preservation of specimens from infectious disease cases	5	4.55	0.59	0.13	60
	B3_6 Ability to correctly carry out epidemiological investigations and write investigation reports	5	4.75	0.43	0.09	75
	B3_7 Analyzing the situation of public health incidents and proposing targeted prevention and control measures	5	4.85	0.36	0.07	85
	B4_1 Ability to undertake research projects independently	4	4.20	0.60	0.14	30
	B4_2 Research design ability	4	4.25	0.43	0.10	25
	B4_3 Ability to write research papers independently	4	4.15	0.48	0.11	20
	B4_4 Understanding domestic and foreign status and trends in the profession	4	4.35	0.65	0.15	45
C Leadership	C1_1 Proper allocation of tasks	5	4.70	0.46	0.10	70
	C1_2 Ability to obtain resources needed for teamwork	4.5	4.45	0.59	0.13	50
	C1_3 Ability to allocate and dispatch resources needed for teamwork	5	4.50	0.67	0.15	60
	C1_4 Supervising and adjusting project implementation	5	4.60	0.58	0.13	65
	C2_1 Systematic understanding of current public health issues	5	4.55	0.50	0.11	55
	C2_2 Integrating different perspectives during decision making	5	4.75	0.43	0.09	75
	C2_3 Clarifying problems to be solved and expected outcomes of decisions	5	4.65	0.48	0.10	65
	C2_4 Ability to formulate alternative plans and select the optimal one	5	4.85	0.36	0.07	85
	C2_5 Ability to execute projects	5	4.75	0.43	0.09	75
	C3_1 Creating an environment conducive to opinion exchange within the department	5	4.55	0.50	0.11	55
	C3_2 Having characteristics to enable effective team leadership like integrity, enthusiasm, honesty, caring, trustworthiness, sense of responsibility, etc	5	4.55	0.59	0.13	60
	C3_3 Establishing effective team motivation models including listening, dialoguing, negotiating, rewarding, encouragement, inspiration, etc	4.5	4.45	0.59	0.13	50
	C4_1 Ability to communicate and coordinate with superiors, subordinates and partners	5	4.60	0.49	0.11	60
	C4_2 Regularly communicating information regarding public health needs, goals, achievements and major crises to target audience through media	4	4.45	0.50	0.11	45
	C4_3 Effectively applying negotiation skills in resolving disputes	4	4.45	0.50	0.11	45
	C5_1 Understanding the impact of own behaviors or reactions on team members	4	4.30	0.64	0.15	40
	C5_2 Giving proper feedback to criticisms on own behaviors or performance by others	4	4.35	0.65	0.15	45
	C5_3 Pressure toughness and ability to deal with complex problems	5	4.50	0.59	0.13	55
	C6_1 Identifying opportunities for team growth, innovation, reform and development	4	4.35	0.65	0.15	45
	C6_2 Creating opportunities for teams to learn and improve together	4	4.30	0.71	0.17	45
	C6_3 Helping members clarify thinking and turn ideas into feasible plans	4	4.40	0.58	0.13	45
	C6_4 Post-incident learning and summarization abilities	5	4.60	0.49	0.11	60
D Personal quality	D1_1 Having the level of education or professional training to meet the requirements of the job	4	4.40	0.58	0.13	45
	D1_2 The major was related to infectious disease prevention and control or health emergency	4	4.15	0.57	0.14	25
	D1_3 Obtaining relevant professional qualification certificates	4	3.85	0.57	0.15	10
	D2_1 Physical fitness	5	4.50	0.67	0.15	60
	D2_2 Psychological quality	5	4.60	0.58	0.13	65
	D2_3 Political literacy	4.5	4.45	0.59	0.13	50
	D2_4 Abiding by the work standard and assuming the work responsibility	5	4.50	0.59	0.13	55
	D2_5 Ability to continue studying and self-improvement	5	4.55	0.59	0.13	60
	D2_6 Understanding my own work role and carry out appropriate work	5	4.50	0.59	0.13	55
	D2_7 Training and guiding ability (guiding students or subordinates)	4	4.35	0.65	0.15	45

*SD* standard deviation, *CV* coefficient of variation

### Evaluation and validation of the scale

#### Participants’ characteristics

A total of 155 subjects were enrolled in the pilot survey for item analysis and EFA, and 476 subjects were enrolled in the formal survey for CFA (Table 4). Age, gender, education level, years of work related to infectious diseases, position, and postgraduate supervisor qualification did not differ statistically between subjects in the two surveys. In contrast, major of bachelor/college, professional title, job type, monthly income, experience in prevention and control of infectious diseases, and number of participations in outbreak response varied between the groups.

**Table 4:** Basic characteristics of participants in the pilot survey and formal survey

Characteristics	Pilot survey		Formal survey		*P*
	Frequency, n	Percentage, %	Frequency, n	Percentage, %	
Age, years					0.376
35–40	75	48.4	234	49.2	
41–45	45	29.0	157	33.0	
46–50	35	22.6	85	17.9	
Gender					0.138
Male	63	40.7	226	47.5	
Female	92	59.4	250	52.5	
Education level					0.340
Doctorate	8	5.2	23	4.8	
Master's Degree	63	40.7	211	44.3	
Bachelor's Degree	84	54.2	234	49.2	
Junior college or below			8	1.7	
Major of bachelor/college					< 0.001
Public health and preventive medicine	93	60.0	364	76.5	
Clinical medicine	20	12.9	54	11.3	
Other medical disciplines	6	3.9	27	5.7	
Public management	5	3.2	12	2.5	
Other management disciplines	3	1.9	3	0.6	
Other	28	18.1	16	3.4	
Professional title					0.023
Senior	36	23.2	68	14.3	
Sub-senior	63	40.7	235	49.4	
Intermediate	56	36.1	173	36.3	
Years of work related to infectious disease prevention and control					0.380
< 5	15	9.7	47	9.9	
5–9	31	20.0	68	14.3	
10–14	50	32.3	155	32.6	
15–19	27	17.4	110	23.1	
≥ 20	32	20.7	96	20.2	
Job type					0.021
Management positions	7	4.5	5	1.1	
Professional technical positions	118	76.1	367	77.1	
Professional technical and management positions	30	19.4	104	21.9	
Position					0.215
Section member	51	32.9	183	38.5	
Deputy section chief and above	104	67.1	293	61.5	
Postgraduate supervisor qualifications					0.451
Doctoral supervisor	17	11.0	37	7.8	
Master’s supervisor	22	14.2	66	13.9	
None	116	74.8	373	78.4	
Net monthly income, RMB					< 0.001
< 5000	20	12.9	49	10.3	
5000–9999	129	83.2	305	64.1	
10000–20000	5	3.2	115	24.2	
20000–30000	1	0.7	7	1.5	
Experience in prevention and control of infectious diseases					
Zika virus disease	7	4.5	66	13.9	0.002
H1N1 Influenza	84	54.2	280	58.8	0.311
Middle East respiratory syndrome	11	7.1	69	14.5	0.016
Coronavirus disease 2019	152	98.1	461	96.9	0.583
Ebola virus disease	8	5.2	56	11.8	0.018
Other	65	41.9	201	42.2	0.949
Number of participations in outbreak response					0.039
0	3	1.9	17	3.6	
1–2	21	13.6	42	8.8	
3–5	33	21.3	83	17.4	
6–10	27	17.4	58	12.2	
> 10	71	45.8	276	58.0	

The comparison of basic characteristics between two groups employed Chi Square test or Fisher’s exact test. Intermediate professional title: professionals with several years of practical experience who have demonstrated competence in their field (ranks 10–8); sub-senior professional title: experienced professionals who have made significant contributions to their discipline and exhibited leadership capabilities (ranks 7–5); senior professional title: the highest level, reserved for exceptional professionals with distinguished accomplishments, recognized expertise, and substantial leadership roles within their organizations or fields (ranks 4–1)

#### Item analysis and EFA results

The group with the lower 27% of total scores had significantly lower mean scores than the group with the upper 27% for all indicators (Supplementary Table 10), indicating a high degree of discriminant validity. Cronbach’s Alpha values of the four primary dimensions exceeded 0.893 in the pilot survey (Supplementary Table 11). The corrected item-total correlation coefficients between individual items and the overall scale score ranged from 0.33 to 0.78, with all *P* < 0.001 (Supplementary Table 12). Cronbach’s Alpha of the total scale remained stable and exceeded 0.98 when any individual item was deleted.

The criteria for conducting EFA were met because the KMO values of four primary dimensions were 0.940, 0.929, 0.943, and 0.868, respectively (all *P* < 0.05). The cumulative variance explanations for the four dimensions were 77.463%, 73.976%, 81.174%, and 68.654%, respectively. Three rounds of EFA and item adjustment were conducted to ensure all items fitted the preset theoretical frame (Supplementary Table 12−20). In summary, four tertiary items were deleted based on item analysis or EFA, two secondary items and two tertiary items were merged based on item analysis or EFA, and one tertiary item was moved to another secondary item based on EFA (Supplementary Table 21). The revised scale after item analysis and EFA comprised four primary items, 14 secondary items, and 64 tertiary items (Supplementary Table 22).

#### CFA results

Cronbach’s Alpha values of the four primary dimensions exceeded 0.898 in the formal survey (Supplementary Table 11). All CR values and AVE values exceeded 0.8 and 0.5, respectively, representing a satisfactory convergent validity (Table 5). The standardized factor loading of the items ranged from 0.630 to 0.977. The path diagrams with standardized factor loadings for the four dimensions are shown in Supplementary Figure 1 to 4. Good discriminant validity was observed in each dimension, with the square root of AVE exceeding the correlation coefficients between factors (Supplementary Table 23−26). Among the seven model fit indices, all four dimensions satisfied at least five criteria, indicating a good fit effectiveness in CFA (Supplementary Table 27).

**Table 5:** Confirmatory factor analysis and convergent validity of the scale

Primary item	Path	Standardized factor loading	SE	*P*	CR	AVE
A Knowledge	A1_1 < ---A1	0.859			0.891	0.674
	A1_2 < ---A1	0.865	0.049	< 0.001		
	A1_3 < ---A1	0.717	0.045	< 0.001		
	A1_4 < ---A1	0.833	0.044	< 0.001		
	A2_1 < ---A2	0.751			0.925	0.675
	A2_2 < ---A2	0.866	0.061	< 0.001		
	A2_3 < ---A2	0.850	0.064	< 0.001		
	A2_4 < ---A2	0.780	0.064	< 0.001		
	A2_5 < ---A2	0.860	0.062	< 0.001		
	A2_6 < ---A2	0.814	0.065	< 0.001		
	A3_1 < ---A3	0.825			0.931	0.729
	A3_2 < ---A3	0.850	0.046	< 0.001		
	A3_3 < ---A3	0.891	0.043	< 0.001		
	A3_4 < ---A3	0.883	0.044	< 0.001		
	A3_5 < ---A3	0.818	0.045	< 0.001		
B Practical skills	B1_1 < ---B1	0.775			0.868	0.569
	B1_2 < ---B1	0.697	0.046	< 0.001		
	B1_3 < ---B1	0.718	0.045	< 0.001		
	B1_4 < ---B1	0.764	0.052	< 0.001		
	B1_5 < ---B1	0.813	0.050	< 0.001		
	B2_1 < ---B2	0.867			0.943	0.733
	B2_2 < ---B2	0.874	0.039	< 0.001		
	B2_3 < ---B2	0.858	0.041	< 0.001		
	B2_4 < ---B2	0.852	0.038	< 0.001		
	B2_5 < ---B2	0.860	0.036	< 0.001		
	B2_6 < ---B2	0.824	0.041	< 0.001		
	B3_1 < ---B3	0.907			0.942	0.766
	B3_2 < ---B3	0.872	0.035	< 0.001		
	B3_3 < ---B3	0.802	0.035	< 0.001		
	B3_4 < ---B3	0.877	0.033	< 0.001		
	B3_5 < ---B3	0.913	0.031	< 0.001		
	B4_1 < ---B4	0.940			0.913	0.778
	B4_2 < ---B4	0.882	0.033	< 0.001		
	B4_3 < ---B4	0.820	0.034	< 0.001		
C Leadership	C1_1 < ---C1	0.924			0.942	0.801
	C1_2 < ---C1	0.837	0.033	< 0.001		
	C1_3 < ---C1	0.905	0.031	< 0.001		
	C1_4 < ---C1	0.912	0.029	< 0.001		
	C2_1 < ---C2	0.811			0.932	0.734
	C2_2 < ---C2	0.881	0.049	< 0.001		
	C2_3 < ---C2	0.906	0.048	< 0.001		
	C2_4 < ---C2	0.885	0.049	< 0.001		
	C2_5 < ---C2	0.795	0.049	< 0.001		
	C3_1 < ---C3	0.889			0.928	0.811
	C3_2 < ---C3	0.901	0.036	< 0.001		
	C3_3 < ---C3	0.912	0.036	< 0.001		
	C4_1 < ---C4	0.839			0.926	0.675
	C4_2 < ---C4	0.727	0.055	< 0.001		
	C4_3 < ---C4	0.837	0.048	< 0.001		
	C4_4 < ---C4	0.850	0.047	< 0.001		
	C4_5 < ---C4	0.827	0.044	< 0.001		
	C4_6 < ---C4	0.844	0.049	< 0.001		
	C5_1 < ---C5	0.902			0.934	0.825
	C5_2 < ---C5	0.910	0.034	< 0.001		
	C5_3 < ---C5	0.913	0.033	< 0.001		
D Personal quality	D1_1 < ---D1	0.977			0.877	0.783
	D1_2 < ---D1	0.782	0.058	< 0.001		
	D2_1 < ---D2	0.630			0.899	0.564
	D2_2 < ---D2	0.722	0.076	< 0.001		
	D2_3 < ---D2	0.732	0.067	< 0.001		
	D2_4 < ---D2	0.818	0.067	< 0.001		
	D2_5 < ---D2	0.793	0.071	< 0.001		
	D2_6 < ---D2	0.866	0.070	< 0.001		
	D2_7 < ---D2	0.666	0.079	< 0.001		

*SE* standard deviation, *CR* composite reliability, *AVE* average variance extracted

### Weight determination of each indicator through AHP

The expert judgment matrixes for the three hierarchies are shown in Supplementary Table 28−46. The consistency ratios were less than 0.1 for all the matrixes, indicating good consistency in each. The combined weight for the four primary items were 0.4064 for Knowledge, 0.2878 for Practical skills, 0.2082 for Leadership, and 0.0981 for Personal quality (Table 6). The secondary items with the top three combined weights were Knowledge of infectious diseases (0.1993), Knowledge of public health emergency management (0.1267), and Public health response to infectious diseases (0.1199). The tertiary items with the top five combined weights were Basic epidemiological knowledge of infectious diseases (0.0823), Criteria for judging aggregates epidemics and outbreaks of infectious diseases (0.0486), Mastering knowledge and skills of personal protection (0.0486), Command, coordination and control of health emergency (0.0413), and Analyzing the situation of public health incidents and proposing targeted prevention and control measures (0.0410). The final versions of the IDCCS in English and Chinese are presented in Supplementary Table 47.

**Table 6:** The weight values of final three-level scale

Index type	Items	Weight	Combined weight	Index type	Items	Weight	Combined weight
Primary items	A	0.4064	0.4064	Tertiary items	B2_4	0.1132	0.0096
	B	0.2878	0.2878		B2_5	0.2441	0.0207
	C	0.2082	0.2082		B2_6	0.1545	0.0131
	D	0.0981	0.0981		B3_1	0.1593	0.0191
Secondary items	A1	0.4904	0.1993		B3_2	0.2427	0.0291
	A2	0.3118	0.1267		B3_3	0.0967	0.0116
	A3	0.1978	0.0804		B3_4	0.1593	0.0191
	B1	0.2130	0.0613		B3_5	0.3420	0.0410
	B2	0.2946	0.0848		B4_1	0.3119	0.0068
	B3	0.4166	0.1199		B4_2	0.1972	0.0043
	B4	0.0757	0.0218		B4_3	0.4908	0.0107
	C1	0.2085	0.0434		C1_1	0.4171	0.0181
	C2	0.2089	0.0435		C1_2	0.1198	0.0052
	C3	0.1417	0.0295		C1_3	0.1935	0.0084
	C4	0.3439	0.0716		C1_4	0.2696	0.0117
	C5	0.0970	0.0202		C2_1	0.0966	0.0042
	D1	0.1998	0.0196		C2_2	0.2092	0.0091
	D2	0.8002	0.0785		C2_3	0.1425	0.0062
Tertiary items	A1_1	0.4129	0.0823		C2_4	0.3425	0.0149
	A1_2	0.2439	0.0486		C2_5	0.2092	0.0091
	A1_3	0.0993	0.0198		C3_1	0.3119	0.0092
	A1_4	0.2439	0.0486		C3_2	0.4915	0.0145
	A2_1	0.2036	0.0258		C3_3	0.1966	0.0058
	A2_2	0.0631	0.0080		C4_1	0.2891	0.0207
	A2_3	0.0852	0.0108		C4_2	0.1522	0.0109
	A2_4	0.1184	0.0150		C4_3	0.1522	0.0109
	A2_5	0.2036	0.0258		C4_4	0.0796	0.0057
	A2_6	0.3260	0.0413		C4_5	0.1103	0.0079
	A3_1	0.3246	0.0261		C4_6	0.2165	0.0155
	A3_2	0.2114	0.0170		C5_1	0.3119	0.0063
	A3_3	0.1443	0.0116		C5_2	0.1980	0.0040
	A3_4	0.2114	0.0170		C5_3	0.4901	0.0099
	A3_5	0.1082	0.0087		D1_1	0.7500	0.0147
	B1_1	0.1419	0.0087		D1_2	0.2500	0.0049
	B1_2	0.0962	0.0059		D2_1	0.1121	0.0088
	B1_3	0.3442	0.0211		D2_2	0.2153	0.0169
	B1_4	0.2088	0.0128		D2_3	0.0917	0.0072
	B1_5	0.2088	0.0128		D2_4	0.1452	0.0114
	B2_1	0.1545	0.0131		D2_5	0.2153	0.0169
	B2_2	0.2441	0.0207		D2_6	0.1452	0.0114
	B2_3	0.0896	0.0076		D2_7	0.0752	0.0059

## Discussion

### Key findings of this study

A key strength of the IDCCS lies in its robust development process, which combined expert consensus through the Delphi method with rigorous statistical validation. The high authority coefficient and opinion coordination coefficient achieved across two Delphi rounds underscore the reliability and representativeness of the scale items. The validation process, including item analysis, EFA, and CFA, demonstrated the IDCCS's strong psychometric properties. Notably, the scale exhibited excellent internal consistency and satisfactory construct validity, with factor loadings, CR, and AVE values all meeting or exceeding standard thresholds. These findings support the reliability and validity of the IDCCS as a measurement tool for infectious disease control competencies.

This study marks a significant advancement in the field of public health competency assessment by developing and validating the IDCCS specifically tailored for public health professionals in China. The IDCCS represents a comprehensive and multidimensional approach to competency evaluation, encompassing four primary dimensions: knowledge, practical skills, leadership, and personal qualities.

### Theoretical underpinnings and dimensionality of the IDCCS

The IDCCS was established based on well-founded theoretical frameworks. Knowledge, practical skills, and personal qualities were derived from the competency iceberg model [19], while leadership stemmed from the public health leadership framework [20]. The iceberg model suggests that hidden factors such as motives, traits, self-image, and social roles are key to understanding competency. Visible competencies, such as knowledge and skills can be readily developed through training and skill-building exercises. However, the invisible competencies are more challenging to assess and cultivate [19]. Thus, more items related to knowledge and practical skills were designed to improve the scale's practicality. The frame of public health leadership was developed through expert consensus to inform a leadership curriculum for public health professionals in Europe [20]. We incorporated most of its domains such as systems thinking, collaborative leadership, leadership and communication, leading change, and organizational learning and development. Political leadership related to European public health governance was excluded. Public health leadership is currently lacking in higher education [33, 34]. The inclusion of this dimension effectively complements the competency measurement for public health professionals, aligning with their work needs and personal development goals.

The IDCCS is the first of its kind, exclusively designed to assess competencies in infectious disease control among public health professionals. Existing public health competency scales often cover a broad range of skills not limited to infectious diseases or focus on medical staff rather than public health professionals. For instance, the Core Competencies for Public Health Professionals developed by the Council on Linkages Between Academia and Public Health Practice provide a foundational framework for generic public health competencies, but it is not specific to infectious disease control [35]. Similarly, the Regional Core Competency Framework for Public Health developed by the WHO exhibited six domains, where only one domain (surveillance and control of risks and threats) was directly related to infectious disease control [12]. Thus, these broader competency models, while valuable for setting macro-level development goals, often lack the specificity required for precise measurement of infectious disease control competencies. IDCCS addresses this gap by providing a specialized tool for assessing competencies in infectious disease control among public health professionals. In contrast to previous scales that primarily focused on the field rescue capabilities of medical staff in managing infectious diseases [16, 25], the IDCCS differs from them in several key aspects. First, the IDCCS is tailored to the Chinese public health system, reflecting the unique structure of China's CDC system. Second, the IDCCS integrates the competency iceberg model with the public health leadership framework, while most previous scales were established without clear theoretical basis. Notably, the incorporation of public health leadership as a competency dimension is a novel and distinctive feature of the IDCCS. While earlier scales concentrated on the clinical aspects of infectious disease management, our scale recognizes the pivotal role of public health leadership in effective disease control strategies.

The results of AHP revealed that knowledge and practical skills were weighted most heavily among the primary dimensions. This highlights the high demand for specialized and technical expertise in infectious disease control. Experts tend to view specialized knowledge and practical abilities as prerequisites, thus assigning them greater weights over leadership and personal qualities when judging competencies. Within the secondary dimensions, knowledge of infectious diseases, knowledge of public health emergency management, and public health response to infectious diseases received the highest weights. This premium on specialized knowledge and practical skills aligns with the competency weights at the primary level, revealing more nuanced demands. Infectious disease knowledge forms the vital theoretical foundation; emergency management represents the capability to respond amidst unpredictable crises; and public health response encompasses the comprehensive expertise to coordinate interventions. Collectively, these secondary competencies underscore the diverse abilities required for real-world infectious disease control. Among the tertiary items, the five with the highest weights all belonged to the knowledge and practical skills dimensions, further validating their significance. The premium placed on self-regulation and communication abilities within the leadership competency dimension highlights the high-pressure nature of infectious disease control work and the critical importance of coordination in outbreak response systems [36]. In the personal quality dimension, professional quality carried a higher weight than professional qualifications, indicating that competency evaluation should focus on applicants' professional sentiments, attitudes, and learning abilities, rather than solely relying on resumes while ignoring character and growth potential.

### Potential applications and impact of the IDCCS

Tailored for the Chinese context, the IDCCS was developed and validated with participants recruited from multiple provinces in China. The Delphi technique employed in its construction ensured the authority and representativeness of the scale's items. Furthermore, the incorporation of the AHP method facilitated the scale's practical operationalization among public health professionals, augmenting its usability and applicability in real-world settings. By combining expert consensus and prioritization methods, the IDCCS effectively balances theoretical rigor with practical considerations. This makes it useful for assessing and nurturing competencies vital to infectious disease control within the Chinese public health system. The IDCCS holds significant potential for practical applications in performance evaluation, recruitment, curriculum development, and self-assessment within public health agencies. During infectious disease outbreaks, the scale can facilitate identifying and deploying highly competent professionals to frontline response efforts and critical incident management. Furthermore, it can serve as a self-assessment tool, promoting intrinsic motivation for learning and professional development among public health professionals. For institutes or agencies, the IDCCS guides the identification of workforce competency gaps, enabling targeted capacity-building initiatives tailored to specific needs and job requirements. By recognizing the varying weights assigned to different competency items, professionals can prioritize and focus on developing the core critical abilities essential for their roles. Moreover, the IDCCS informs the design and evaluation of training programs and simulation exercises, providing a theoretical framework for assessing their effectiveness.

While the IDCCS was developed within the Chinese context, its framework and methodology have potential global applications. For adaptation to other regions, we have several recommendations. First, the core dimensions (knowledge, practical skills, leadership, personal qualities) are suggested to be retained while adjusting specific indicators to reflect local public health systems and infectious disease control priorities. Second, new Delphi processes are suggested to be conducted through engaging local public health experts to refine indicators and recalibrate weights according to regional needs and perspectives. Third, new indicators related to local cultural contexts, policy environments, and specific infectious disease challenges should be incorporated. Additionally, cross-cultural validity testing is necessary to ensure the adapted scale's reliability and validity in the new context [37].

### Limitations

This study has several limitations that should be considered. Methodologically, the Delphi process excluded health officials and administrators, which may have limited the scale's reflection of implementation and policy perspectives. The competency evaluation relied on self-reported assessments, which may introduce leniency bias. In terms of scope and applicability, the IDCCS was specifically developed for public health professionals in infectious disease control within the CDC system, potentially limiting its suitability for evaluating professionals beyond this context or in other public health domains. Geographically and culturally, our study sample was limited to multiple provinces in China, which may restrict the scale's applicability to regions with distinct cultural, socioeconomic, and public health system characteristics. Future research should address these limitations by including a broader range of stakeholders in the development process, incorporating objective assessment methods, and exploring the scale's cross-cultural validity and potential adaptations required for broader national or international applications.

## Conclusions

This study has established, for the first time, a competency framework tailored specifically for public health professionals in infectious disease control. The IDCCS covers a comprehensive spectrum of competencies, addressing knowledge, practical skills, leadership, and personal qualities. It holds significant potential for application in performance evaluation, recruitment processes, curriculum development, and individual self-assessment within public health agencies. While providing crucial support in addressing key public health issues, the scale requires practical validation to continually enhance and optimize its utility.

## Supplementary Information

Supplementary file 1

## Data Availability

The datasets used and/or analyzed during the current study are available from the corresponding author upon reasonable request.
