# Supplementary material for: Development and validation of an infectious disease control competency scale for public health professionals
Source: Glob Health Res Policy. 2024 Sep 26;9:39. doi: 10.1186/s41256-024-00381-y (PMC11426014; doi:10.1186/s41256-024-00381-y)
Supplement: Supplementary file 1 [file 41256_2024_381_MOESM1_ESM.docx]

**Supplementary materials**

**Contents**

[Supplementary Table 1. The preliminary version of the scale 4](#_Toc166526752)

[Supplementary Table 2. The authority degree of experts in the first round of Delphi 9](#_Toc166526753)

[Supplementary Table 3. The revision methods of items not meeting the threshold values or revised by experts in the first round of Delphi 10](#_Toc166526754)

[Supplementary Table 4. Kendall’s W statistic of the first round of Delphi 14](#_Toc166526755)

[Supplementary Table 5. Revised scale after the first round of Delphi 15](#_Toc166526756)

[Supplementary Table 6. The authority degree of experts in the second round of Delphi 19](#_Toc166526757)

[Supplementary Table 7. The revision methods of items not meeting the threshold values or revised by experts in the second round of Delphi 20](#_Toc166526758)

[Supplementary Table 8. Kendall’s W statistic of the first round of Delphi 23](#_Toc166526759)

[Supplementary Table 9. Revised scale after the second round of Delphi 24](#_Toc166526760)

[Supplementary Table 10. Score comparison between high-score and low-score groups 30](#_Toc166526761)

[Supplementary Table 11. Results of Cronbach's Alpha of the scale based on the pilot survey and the formal survey 34](#_Toc166526762)

[Supplementary Table 12. Inspection results of scale items 35](#_Toc166526763)

[Supplementary Table 13. Factor loadings of items in the knowledge section (first round of exploratory factor analysis) 38](#_Toc166526764)

[Supplementary Table 14. Factor loadings of items in the section of practical skills (first round of exploratory factor analysis) 39](#_Toc166526765)

[Supplementary Table 15. Factor loadings of items in the leadership section (first round of exploratory factor analysis) 41](#_Toc166526766)

[Supplementary Table 16. Factor loadings of items in the section of personal quality 42](#_Toc166526767)

[Supplementary Table 17. Factor loadings of items in the knowledge section (second round of exploratory factor analysis) 43](#_Toc166526768)

[Supplementary Table 18. Factor loadings of items in the section of practical skills (second round of exploratory factor analysis) 44](#_Toc166526769)

[Supplementary Table 19. Factor loadings of items in the leadership section (second round of exploratory factor analysis) 45](#_Toc166526770)

[Supplementary Table 20. Factor loadings of items in the leadership section (third round of exploratory factor analysis) 47](#_Toc166526771)

[Supplementary Table 21. The revision methods of items not passing the item analysis or EFA 49](#_Toc166526772)

[Supplementary Table 22. Revised scale after item analysis and exploratory factor analysis 50](#_Toc166526773)

[Supplementary Figure 1. Path diagram for knowledge section derived by confirmatory factor analysis. 54](#_Toc166526774)

[Supplementary Figure 2. Path diagram for the section of practical skills derived by confirmatory factor analysis. 55](#_Toc166526775)

[Supplementary Figure 3. Path diagram for the leadership section derived by confirmatory factor analysis. 56](#_Toc166526776)

[Supplementary Figure 4. Path diagram for the section of personal quality derived by confirmatory factor analysis. 57](#_Toc166526777)

[Supplementary Table 23. Discriminant validity of the knowledge section 58](#_Toc166526778)

[Supplementary Table 24. Discriminant validity of the section of practical skills 58](#_Toc166526779)

[Supplementary Table 25. Discriminant validity of the leadership section 59](#_Toc166526780)

[Supplementary Table 26. Discriminant validity of the section of personal quality 59](#_Toc166526781)

[Supplementary Table 27. Model fit of four primary sections 60](#_Toc166526782)

[Supplementary Table 28. Judgment matrix and consistency test of the primary items 60](#_Toc166526783)

[Supplementary Table 29. Judgment matrix and consistency test of the knowledge section 61](#_Toc166526784)

[Supplementary Table 30. Judgment matrix and consistency test of the section of practical skills 61](#_Toc166526785)

[Supplementary Table 31. Judgment matrix and consistency test of the leadership section 61](#_Toc166526786)

[Supplementary Table 32. Judgment matrix and consistency test of the section of personal quality 62](#_Toc166526787)

[Supplementary Table 33. Judgment matrix and consistency test of A1 Knowledge of infectious diseases 62](#_Toc166526788)

[Supplementary Table 34. Judgment matrix and consistency test of A2 Knowledge of public health emergency management 62](#_Toc166526789)

[Supplementary Table 35. Judgment matrix and consistency test of A3 Laws, plans and mechanisms for responding to public health emergencies 63](#_Toc166526790)

[Supplementary Table 36. Judgment matrix and consistency test of B1 Infectious diseases prevention and emergency preparedness 63](#_Toc166526791)

[Supplementary Table 37. Judgment matrix and consistency test of B2 Infectious diseases surveillance and early warning 64](#_Toc166526792)

[Supplementary Table 38. Judgment matrix and consistency test of B3 Public health response to infectious diseases 64](#_Toc166526793)

[Supplementary Table 39. Judgment matrix and consistency test of B4 Scientific research ability 65](#_Toc166526794)

[Supplementary Table 40. Judgment matrix and consistency test of C1 Leadership fundamentals 65](#_Toc166526795)

[Supplementary Table 41. Judgment matrix and consistency test of C2 Decision-making ability 65](#_Toc166526796)

[Supplementary Table 42. Judgment matrix and consistency test of C3 Team mobilization ability 66](#_Toc166526797)

[Supplementary Table 43. Judgment matrix and consistency test of C4 Self-regulation and communication abilities 66](#_Toc166526798)

[Supplementary Table 44. Judgment matrix and consistency test of C5 Team learning and development 66](#_Toc166526799)

[Supplementary Table 45. Judgment matrix and consistency test of D1 Professional qualifications 67](#_Toc166526800)

[Supplementary Table 46. Judgment matrix and consistency test of D2 Professional quality 67](#_Toc166526801)

[Supplementary Table 47. Final scale in English and Chinese 68](#_Toc166526802)

Supplementary Table 1. The preliminary version of the scale

| **Primary items** | **Secondary items** | **Tertiary items** |
| --- | --- | --- |
|  |  |  |
|  |  |  |
| **A Knowledge** | A1 Basic knowledge of infectious diseases | A1_1 Pathogenic biology of common infectious disease pathogens |
|  |  | A1_2 Criteria for judging infectious source of infectious diseases |
|  |  | A1_3 Criteria for judging transmission route of pathogens |
|  |  | A1_4 Susceptible populations for common infectious diseases |
|  |  | A1_5 Criteria for judging aggregates epidemics and outbreaks of infectious diseases |
|  |  | A1_6 Influencing factors of epidemic spread of infectious diseases |
|  |  | A1_7 Common prevention and control measures for infectious diseases |
|  |  | A1_8 Clinical manifestations of common infectious diseases |
|  |  | A1_9 Diagnostic criteria and differential diagnosis of common infectious diseases |
|  |  | A1_10 Treatment principles of common infectious diseases |
|  | A2 Basic knowledge of public health emergency management | A2_1 Fundamentals of public health emergency management |
|  |  | A2_2 Classification of infectious diseases surveillance |
|  |  | A2_3 Steps of early warning of public health emergency |
|  |  | A2_4 Theories of health emergency management |
|  |  | A2_5 Theories of crisis decision making |
|  |  | A2_6 Theories of risk assessment |
|  |  | A2_7 Theories of risk communication |
|  |  | A2_8 Command, coordination and control of health emergency |
|  | A3 National plan and systems for public health emergencies in China (emergency plan, emergency management system, operation mechanism and legal system) | A3_1 Responsibilities of disease control personnel in National Emergency Response Plan for Public Health Emergencies |
|  |  | A3_2 Responsibilities of disease control personnel in National Medical Rescue Scheme for Public Health Emergencies |
|  |  | A3_3 Understanding of responsibilities of disease control personnel in Law on Prevention and Control of Infectious Diseases of the People's Republic of China |
|  |  | A3_4 Responsibilities of disease control personnel in Regulations on Emergency Response to Public Health Emergencies |
|  |  | A3_5 Prevention and preparedness mechanism for public health emergencies in China |
|  |  | A3_6 Surveillance and early warning mechanism for public health emergencies in China |
|  |  | A3_7 Response and rescue mechanism for public health emergencies in China |
|  |  | A3_8 Aftermath assessment mechanism for public health emergencies in China |
|  |  | A3_9 Health emergency system in China |
| **B Practical skills** | B1 Infectious diseases prevention and emergency preparedness | B1_1 Development of emergency plans |
|  |  | B1_2 Health promotion |
|  |  | B1_3 Receiving professional training |
|  |  | B1_4 Participating in emergency drills |
|  |  | B1_5 Emergency capacity assessment |
|  | B2 Infectious diseases surveillance and early warning | B2_1 Clarifying the content and process of infectious diseases surveillance |
|  |  | B2_2 Ability to detect abnormal signals of public health emergencies |
|  |  | B2_3 Clarifying the reporting process for public health emergencies |
|  |  | B2_4 Determining reliability of information sources for emerging infectious diseases |
|  |  | B2_5 Ability to extract key information from selected information sources |
|  |  | B2_6 Predicting occurrence and epidemic trends of infectious diseases based on surveillance information |
|  | B3 Public health response to infectious diseases | B3_1 Mastering knowledge and skills of personal protection |
|  |  | B3_2 Mastering principles of setting up isolation wards for infectious diseases |
|  |  | B3_3 Ability to properly handle items or corpses involving infectious pathogens |
|  |  | B3_4 Mastering methods of environmental disinfection and sampling |
|  |  | B3_5 Correctly implementing isolation measures for various infectious disease patients |
|  |  | B3_6 Ability to correctly carry out epidemiological investigations and write investigation reports |
|  |  | B3_7 Analyzing the situation of public health incidents and proposing targeted prevention and control measures |
|  |  | B3_8 Clarifying on-site processing procedures for public health emergencies |
|  | B4 Scientific research ability | B4_1 Having honorary titles of expert |
|  |  | B4_2 Undertaking research projects |
|  |  | B4_3 Publishing research papers |
|  |  | B4_4 Authoring professional publications |
|  |  | B4_5 Obtaining national patents |
|  |  | B4_6 Winning research awards |
|  |  | B4_7 Research design ability |
|  |  | B4_8 Chinese paper writing ability |
|  |  | B4_9 English paper writing ability |
|  |  | B4_10 Understanding domestic and foreign status and trends in the profession |
|  | B5 Business guidance ability | B5_1 Experience in guiding students |
|  |  | B5_2 Experience in guiding subordinate in training and learning |
|  |  | B5_3 Training guidance ability |
| **C Leadership** | C1 Leadership fundamentals | C1_1 Task assignment with division of responsibilities |
|  |  | C1_2 Ability to obtain resources needed for teamwork |
|  |  | C1_3 Ability to allocate and dispatch resources needed for teamwork |
|  |  | C1_4 Supervising and adjusting project implementation |
|  | C2 Decision-making ability | C2_1 Systematic understanding of current public health issues |
|  |  | C2_2 Integrating different perspectives during decision making |
|  |  | C2_3 Clarifying problems to be solved and expected outcomes of decisions |
|  |  | C2_4 Ability to formulate alternative plans and select the optimal one |
|  |  | C2_5 Ability to execute project plans |
|  | C3 Team mobilization ability | C3_1 Creating an environment conducive to opinion exchange within the department |
|  |  | C3_2 Having characteristics to enable effective team leadership like integrity, enthusiasm, honesty, caring, trustworthiness, sense of responsibility, etc. |
|  |  | C3_3 Establishing effective team motivation models including listening, dialoguing, negotiating, rewarding, encouragement, inspiration, etc. |
|  | C4 Communication skills | C4_1 Demonstrating excellent writing, communication and presentation skills |
|  |  | C4_2 Regularly communicating information regarding public health needs, goals, achievements and major crises to target audience through media |
|  |  | C4_3 Effectively applying negotiation skills in resolving disputes |
|  | C5 Self-regulation and interpersonal coordination abilities | C5_1 Understanding the impact of own behaviors or reactions on team members |
|  |  | C5_2 Giving proper feedback to criticisms on own behaviors or performance by others |
|  |  | C5_3 Demonstrating adaptability and ability to motivate myself when facing threats or pressure |
|  | C6 Team learning and development | C6_1 Identifying opportunities for team growth, innovation, reform and development |
|  |  | C6_2 Creating opportunities for teams to learn and improve together |
|  |  | C6_3 Helping members clarify thinking and turn ideas into feasible plans |
|  |  | C6_4 Post-incident learning and summarization abilities |

Supplementary Table 2. The authority degree of experts in the first round of Delphi

| **Expert number** | **Knowledge section** | | |  | **Section of practical skills** | | |  | **Leadership section** | | |
| --- | --- | --- | --- | --- | --- | --- | --- | --- | --- | --- | --- |
|  | **Cs** | **Ca** | **Cr** |  | **Cs** | **Ca** | **Cr** |  | **Cs** | **Ca** | **Cr** |
| 1 | 1 | 1 | 1.00 |  | 1 | 0.9 | 0.95 |  | 0.6 | 0.9 | 0.75 |
| 2 | 0.8 | 1 | 0.90 |  | 0.8 | 1 | 0.90 |  | 0.8 | 0.9 | 0.85 |
| 3 | 1 | 0.9 | 0.95 |  | 1 | 1 | 1.00 |  | 1 | 0.9 | 0.95 |
| 4 | 1 | 0.9 | 0.95 |  | 1 | 0.9 | 0.95 |  | 1 | 0.8 | 0.90 |
| 5 | 1 | 1 | 1.00 |  | 1 | 1 | 1.00 |  | 1 | 1 | 1.00 |
| 6 | 1 | 1 | 1.00 |  | 1 | 1 | 1.00 |  | 1 | 1 | 1.00 |
| 7 | 0.8 | 0.9 | 0.85 |  | 0.8 | 0.9 | 0.85 |  | 1 | 0.9 | 0.95 |
| 8 | 1 | 0.9 | 0.95 |  | 1 | 0.9 | 0.95 |  | 1 | 1 | 1.00 |
| 9 | 0.8 | 1 | 0.90 |  | 0.8 | 1 | 0.90 |  | 0.8 | 1 | 0.90 |
| 10 | 1 | 0.9 | 0.95 |  | 1 | 0.9 | 0.95 |  | 1 | 1 | 1.00 |
| 11 | 1 | 1 | 1.00 |  | 1 | 1 | 1.00 |  | 0.8 | 0.9 | 0.85 |
| 12 | 1 | 1 | 1.00 |  | 1 | 0.9 | 0.95 |  | 1 | 0.8 | 0.90 |
| 13 | 1 | 1 | 1.00 |  | 1 | 1 | 1.00 |  | 1 | 1 | 1.00 |
| 14 | 0.8 | 0.9 | 0.85 |  | 0.8 | 0.9 | 0.85 |  | 0.8 | 1 | 0.90 |
| 15 | 0.8 | 1 | 0.90 |  | 0.8 | 1 | 0.90 |  | 0.6 | 0.8 | 0.70 |
| 16 | 1 | 1 | 1.00 |  | 1 | 1 | 1.00 |  | 1 | 1 | 1.00 |
| 17 | 1 | 1 | 1.00 |  | 1 | 1 | 1.00 |  | 1 | 1 | 1.00 |
| 18 | 1 | 0.8 | 0.90 |  | 0.8 | 0.8 | 0.80 |  | 0.8 | 0.9 | 0.85 |
| 19 | 0.6 | 1 | 0.80 |  | 0.8 | 0.9 | 0.85 |  | 0.8 | 0.9 | 0.85 |
| 20 | 0.8 | 0.8 | 0.80 |  | 0.8 | 0.9 | 0.85 |  | 0.8 | 0.8 | 0.80 |
| **Mean** | **0.920** | **0.950** | **0.935** |  | **0.920** | **0.945** | **0.933** |  | **0.890** | **0.925** | **0.908** |

Note: Cs, familiarity degree; Ca, judgment coefficient; Cr, authority coefficient.

Supplementary Table 3. The revision methods of items not meeting the threshold values or revised by experts in the first round of Delphi

| Original items | Reasons for revision | Revision methods |
| --- | --- | --- |
| - | Experts suggested adding two items (professional ethics and self-learning ability). | A new primary item named "Personal quality" was added, including two secondary items (professional qualifications and professional quality). |
| A1 Basic knowledge of infectious diseases | Experts suggested that basic knowledge was too simple for core public health workforce. | Revised to "Knowledge of infectious diseases" |
| A2 Basic knowledge of public health emergency management | Experts suggested that basic knowledge is too simple for core public health workforce. | Revised to "Knowledge of public health emergency management" |
| A3 National plan and systems for public health emergencies in China (emergency plan, emergency management system, operation mechanism and legal system) | Experts suggested that this item should be simplified. | Revised to "Laws, plans and mechanisms for responding to public health emergencies" |
| B4 Scientific research ability | The mean score was below the boundary of 4.05, requiring adjustment by the research team. | This item was retained because improving the scientific research ability of core public health workforce is the future policy direction. |
| B5 Business guidance ability | It was more appropriate to be included by primary item "Personal quality". | Delete this secondary item, and move its included tertiary items under the secondary item of professional quality. |
| A1_1 Pathogenic biology of common infectious disease pathogens A1_2 Criteria for judging infectious source of infectious diseases A1_3 Criteria for judging transmission route of pathogens A1_4 Susceptible populations for common infectious diseases | Experts suggested merging the four items into one item. | The four items were merge into one item "Basic knowledge of infectious diseases (etiology knowledge, judgment of infection source and transmission route, understanding of susceptible population, etc.)". |
| A1_10 Treatment principles of common infectious diseases | The mean score was below the boundary of 4.05, requiring adjustment by the research team. | Deleted. |
| A2_2 Classification of infectious disease surveillance | Not accurate. | Revised to "Different ways of infectious disease surveillance" |
| A3_1 Responsibilities of disease control personnel in National Emergency Response Plan for Public Health Emergencies A3_2 Responsibilities of disease control personnel in National Medical Rescue Scheme for Public Health Emergencies A3_3 Understanding of responsibilities of disease control personnel in Law on Prevention and Control of Infectious Diseases of the People's Republic of China A3_4 Responsibilities of disease control personnel in Regulations on Emergency Response to Public Health Emergencies | The mean score was below the boundary of 4.05, and these items were suggested to be merged by experts. | The four items were merge into two items "The knowledge of relevant laws and regulations on infectious disease prevention and control and health emergency in China" and "The knowledge of the prevention and control of infectious diseases and related health emergency plans in China" |
| A3_8 Aftermath assessment mechanism for public health emergencies in China | The mean score was below the boundary of 4.05, requiring adjustment by the research team. | Deleted. |
| A3_9 Health emergency system in China | The mean score was below the boundary of 4.05, requiring adjustment by the research team. | Deleted. |
| B1_2 Health promotion | Not accurate. | Revised to "Health popularization on infectious diseases and public health emergencies". |
| B2_4 Determining reliability of information sources for emerging infectious diseases | The mean score was below the boundary of 4.05, requiring adjustment by the research team. | Revised to "Determining the reliability of information sources on infectious diseases". |
| B3_2 Mastering principles of setting up isolation wards for infectious diseases | Experts suggested placing this item with "Master the principles of defining epidemic areas". | Revised to "Mastering the principles of defining epidemic areas". |
| B3_3 Ability to properly handle items or corpses involving infectious pathogens B3_4 Mastering methods of environmental disinfection and sampling B3_5 Correctly implementing isolation measures for various infectious disease patients | Experts recommend merging these three items because they are too specific. | Revised to "Familiar with the methods of case management and disinfection in the field environment". |
| - | Experts suggested adding one item (Correctly implementing the collection, transportation and preservation of specimens from infectious disease cases) under B3 Public health response to infectious diseases. | A new item "Correctly implementing the collection, transportation and preservation of specimens from infectious disease cases" was added under B3. |
| B4_1 Having honorary titles of expert B4_2 Undertaking research projects B4_3 Publishing research papers B4_4 Authoring professional publications B4_5 Obtaining national patents B4_6 Winning research awards | The mean score was below the boundary of 4.05, requiring adjustment by the research team. | These items were merged into one item "Ability to undertake research projects independently". |
| B4_8 Chinese paper writing ability B4_9 English paper writing ability | The mean score was below the boundary of 4.05, requiring adjustment by the research team. | These items were merged into one item "Ability to write research papers independently". |
| B5_1 Experience in guiding students B5_2 Experience in guiding subordinate in training and learning B5_3 Training guidance ability | The mean score was below the boundary of 4.05, and experts suggested that there was duplication in the content of these three items. | These items were merged into one item "Training and guiding ability (guiding students or subordinates)", and moved under the primary item "Personal quality". |
| C1_1 Task assignment with division of responsibilities | Not accurate. | Revised to "Proper allocation of tasks". |
| C2_5 Ability to execute project plans | Not accurate. | Revised to "Ability to execute projects". |
| C4_1 Demonstrating excellent writing, communication and presentation skills | The mean score was below the boundary of 4.05, requiring adjustment by the research team. | Revised to "Ability to communicate and coordinate with superiors, subordinates and partners". |
| C5_3 Demonstrating adaptability and ability to motivate myself when facing threats or pressure | The mean score was below the boundary of 4.05, requiring adjustment by the research team. | Revised to "Pressure toughness and ability to deal with complex problems". |
| - | A new primary item named "Personal quality" was added, including two secondary items (professional qualifications and professional quality) | Adding items: Professional qualifications: 1. Having the level of education or professional training to meet the requirements of the job 2. The major was related to infectious disease prevention and control or health emergency 3. Obtaining relevant professional qualification certificates  Professional quality 1. Physical fitness 2. Psychological quality 3. Political literacy 4. Abiding by the work standard and assuming the work responsibility 5. Ability to continue studying and self-improvement 6. Understanding my own work role and carry out appropriate work 7. Training and guiding ability (guiding students or subordinates) |

Supplementary Table 4. Kendall’s W statistic of the first round of Delphi

| **W** | **χ²** | ***P*** |
| --- | --- | --- |
| 0.285 | 594.724 | < 0.001 |

Supplementary Table 5. Revised scale after the first round of Delphi

| Primary items | Secondary items | Tertiary items |
| --- | --- | --- |
|  |  |  |
|  |  |  |
| A Knowledge | A1 Knowledge of infectious diseases | A1_1 Basic knowledge of infectious diseases (etiology knowledge, judgment of infection source and transmission route, understanding of susceptible population, etc.) |
|  |  | A1_2 Criteria for judging aggregates epidemics and outbreaks of infectious diseases (including nosocomial infections) |
|  |  | A1_3 Influencing factors of epidemic spread of infectious diseases |
|  |  | A1_4 Common prevention and control measures for infectious diseases |
|  |  | A1_5 Clinical manifestations of common infectious diseases |
|  |  | A1_6 Diagnostic criteria and differential diagnosis of common infectious diseases |
|  | A2 Knowledge of public health emergency management | A2_1 Fundamentals of public health emergency management |
|  |  | A2_2 Different ways of infectious disease surveillance |
|  |  | A2_3 Steps of early warning of public health emergency |
|  |  | A2_4 Theories of health emergency management |
|  |  | A2_5 Theories of crisis decision making |
|  |  | A2_6 Theories of risk assessment |
|  |  | A2_7 Theories of risk communication |
|  |  | A2_8 Command, coordination and control of health emergency |
|  | A3 Laws, plans and mechanisms for responding to public health emergencies | A3_1 The knowledge of relevant laws and regulations on infectious disease prevention and control and health emergency in China |
|  |  | A3_2 The knowledge of the prevention and control of infectious diseases and related health emergency plans in China |
|  |  | A3_3 Prevention and preparedness mechanism for public health emergencies in China |
|  |  | A3_4 Surveillance and early warning mechanism for public health emergencies in China |
|  |  | A3_5 Response and rescue mechanism for public health emergencies in China |
| B Practical skills | B1 Infectious diseases prevention and emergency preparedness | B1_1 Development of emergency plans |
|  |  | B1_2 Health popularization on infectious diseases and public health emergencies |
|  |  | B1_3 Receiving professional training |
|  |  | B1_4 Participating in emergency drills |
|  |  | B1_5 Emergency capacity assessment |
|  | B2 Infectious diseases surveillance and early warning | B2_1 Clarifying the content and process of infectious diseases surveillance |
|  |  | B2_2 Ability to detect abnormal signals of public health emergencies |
|  |  | B2_3 Clarifying the reporting process for public health emergencies |
|  |  | B2_4 Determining the reliability of information sources on infectious diseases |
|  |  | B2_5 Ability to extract key information from selected information sources |
|  |  | B2_6 Predicting occurrence and epidemic trends of infectious diseases based on surveillance information |
|  | B3 Public health response to infectious diseases | B3_1 Mastering knowledge and skills of personal protection |
|  |  | B3_2 Clarifying on-site processing procedures for public health emergencies |
|  |  | B3_3 Mastering the principles of defining epidemic areas |
|  |  | B3_4 Familiar with the methods of case management and disinfection in the field environment |
|  |  | B3_5 Correctly implementing the collection, transportation and preservation of specimens from infectious disease cases |
|  |  | B3_6 Ability to correctly carry out epidemiological investigations and write investigation reports |
|  |  | B3_7 Analyzing the situation of public health incidents and proposing targeted prevention and control measures |
|  | B4 Scientific research ability | B4_1 Ability to undertake research projects independently |
|  |  | B4_2 Research design ability |
|  |  | B4_3 Ability to write research papers independently |
|  |  | B4_4 Understanding domestic and foreign status and trends in the profession |
| C Leadership | C1 Leadership fundamentals | C1_1 Proper allocation of tasks |
|  |  | C1_2 Ability to obtain resources needed for teamwork |
|  |  | C1_3 Ability to allocate and dispatch resources needed for teamwork |
|  |  | C1_4 Supervising and adjusting project implementation |
|  | C2 Decision-making ability | C2_1 Systematic understanding of current public health issues |
|  |  | C2_2 Integrating different perspectives during decision making |
|  |  | C2_3 Clarifying problems to be solved and expected outcomes of decisions |
|  |  | C2_4 Ability to formulate alternative plans and select the optimal one |
|  |  | C2_5 Ability to execute projects |
|  | C3 Team mobilization ability | C3_1 Creating an environment conducive to opinion exchange within the department |
|  |  | C3_2 Having characteristics to enable effective team leadership like integrity, enthusiasm, honesty, caring, trustworthiness, sense of responsibility, etc. |
|  |  | C3_3 Establishing effective team motivation models including listening, dialoguing, negotiating, rewarding, encouragement, inspiration, etc. |
|  | C4 Communication skills | C4_1 Ability to communicate and coordinate with superiors, subordinates and partners |
|  |  | C4_2 Regularly communicating information regarding public health needs, goals, achievements and major crises to target audience through media |
|  |  | C4_3 Effectively applying negotiation skills in resolving disputes |
|  | C5 Self-regulation and interpersonal coordination abilities | C5_1 Understanding the impact of own behaviors or reactions on team members |
|  |  | C5_2 Giving proper feedback to criticisms on own behaviors or performance by others |
|  |  | C5_3 Pressure toughness and ability to deal with complex problems |
|  | C6 Team learning and development | C6_1 Identifying opportunities for team growth, innovation, reform and development |
|  |  | C6_2 Creating opportunities for teams to learn and improve together |
|  |  | C6_3 Helping members clarify thinking and turn ideas into feasible plans |
|  |  | C6_4 Post-incident learning and summarization abilities |
| D Personal quality | D1 Professional qualifications | D1_1 Having the level of education or professional training to meet the requirements of the job |
|  |  | D1_2 The major was related to infectious disease prevention and control or health emergency |
|  |  | D1_3 Obtaining relevant professional qualification certificates |
|  | D2 Professional quality | D2_1 Physical fitness |
|  |  | D2_2 Psychological quality |
|  |  | D2_3 Political literacy |
|  |  | D2_4 Abiding by the work standard and assuming the work responsibility |
|  |  | D2_5 Ability to continue studying and self-improvement |
|  |  | D2_6 Understanding my own work role and carry out appropriate work |
|  |  | D2_7 Training and guiding ability (guiding students or subordinates) |

Supplementary Table 6. The authority degree of experts in the second round of Delphi

| **Expert number** | **Knowledge section** | | |  | **Section of practical skills** | | |  | **Leadership section** | | |  | **Section of personal quality** | | |
| --- | --- | --- | --- | --- | --- | --- | --- | --- | --- | --- | --- | --- | --- | --- | --- |
|  | **Cs** | **Ca** | **Cr** |  | **Cs** | **Ca** | **Cr** |  | **Cs** | **Ca** | **Cr** |  | **Cs** | **Ca** | **Cr** |
| 1 | 1 | 0.9 | 0.95 |  | 1 | 1 | 1.00 |  | 1 | 0.9 | 0.95 |  | 1 | 0.9 | 0.95 |
| 2 | 1 | 0.9 | 0.95 |  | 1 | 0.9 | 0.95 |  | 1 | 0.9 | 0.95 |  | 1 | 1 | 1.00 |
| 3 | 1 | 1 | 1.00 |  | 1 | 1 | 1.00 |  | 1 | 1 | 1.00 |  | 1 | 1 | 1.00 |
| 4 | 1 | 0.9 | 0.95 |  | 1 | 0.9 | 0.95 |  | 1 | 1 | 1.00 |  | 1 | 1 | 1.00 |
| 5 | 0.8 | 0.9 | 0.85 |  | 0.8 | 0.9 | 0.85 |  | 0.8 | 0.9 | 0.85 |  | 1 | 1 | 1.00 |
| 6 | 1 | 0.9 | 0.95 |  | 1 | 0.9 | 0.95 |  | 1 | 0.9 | 0.95 |  | 1 | 0.9 | 0.95 |
| 7 | 1 | 1 | 1.00 |  | 1 | 1 | 1.00 |  | 1 | 1 | 1.00 |  | 1 | 1 | 1.00 |
| 8 | 0.8 | 1 | 0.90 |  | 0.8 | 1 | 0.90 |  | 0.8 | 1 | 0.90 |  | 0.8 | 1 | 0.90 |
| 9 | 1 | 1 | 1.00 |  | 1 | 1 | 1.00 |  | 1 | 1 | 1.00 |  | 1 | 1 | 1.00 |
| 10 | 1 | 1 | 1.00 |  | 1 | 1 | 1.00 |  | 1 | 1 | 1.00 |  | 1 | 1 | 1.00 |
| 11 | 0.8 | 0.9 | 0.85 |  | 0.8 | 0.9 | 0.85 |  | 1 | 1 | 1.00 |  | 1 | 1 | 1.00 |
| 12 | 1 | 1 | 1.00 |  | 1 | 1 | 1.00 |  | 0.8 | 0.9 | 0.85 |  | 0.8 | 1 | 0.90 |
| 14 | 0.8 | 0.9 | 0.85 |  | 0.8 | 0.9 | 0.85 |  | 0.8 | 1 | 0.90 |  | 0.8 | 0.9 | 0.85 |
| 15 | 0.8 | 0.9 | 0.85 |  | 0.6 | 0.8 | 0.70 |  | 0.6 | 0.8 | 0.70 |  | 0.8 | 0.8 | 0.80 |
| 16 | 0.8 | 1 | 0.90 |  | 0.8 | 1 | 0.90 |  | 0.8 | 0.9 | 0.85 |  | 0.8 | 1 | 0.90 |
| 17 | 0.8 | 1 | 0.90 |  | 0.8 | 1 | 0.90 |  | 0.8 | 1 | 0.90 |  | 0.8 | 1 | 0.90 |
| 18 | 0.8 | 1 | 0.90 |  | 0.8 | 0.9 | 0.85 |  | 0.8 | 0.9 | 0.85 |  | 0.6 | 0.9 | 0.75 |
| 19 | 1 | 0.9 | 0.95 |  | 1 | 0.9 | 0.95 |  | 1 | 1 | 1.00 |  | 1 | 1 | 1.00 |
| 20 | 1 | 1 | 1.00 |  | 1 | 1 | 1.00 |  | 1 | 1 | 1.00 |  | 1 | 1 | 1.00 |
| 21 | 1 | 1 | 1.00 |  | 0.8 | 1 | 0.90 |  | 1 | 1 | 1.00 |  | 1 | 1 | 1.00 |
| **Mean** | **0.920** | **0.955** | **0.938** |  | **0.900** | **0.950** | **0.925** |  | **0.910** | **0.955** | **0.933** |  | **0.920** | **0.970** | **0.945** |

Note: Cs, familiarity degree; Ca, judgment coefficient; Cr, authority coefficient.

Supplementary Table 7. The revision methods of items not meeting the threshold values or revised by experts in the second round of Delphi

| Original items | Reasons for revision | Revision methods |
| --- | --- | --- |
| D Personal quality | One expert suggested deleting this primary item because it was not relevant to the standards of core public health workforce. | It was retained after discussion because the breakdown items under "personal quality" were important for measuring internal psychological characteristics. |
| B4 Scientific research ability | None of the three indicators reached the boundary values, requiring adjustment by the research team. | It was retained after discussion because improving the ability of scientific research is the requirement put forward by the Chinese government for core public health workforce. |
| D1 Professional qualifications | The arithmetic mean and full mark rate did not reach the boundary values, requiring adjustment by the research team. | It was retained after discussion because the mean of 4 was acceptable, but the tertiary items under it was revised. |
| A1_1 Basic knowledge of infectious diseases (etiology knowledge, judgment of infection source and transmission route, understanding of susceptible population, etc.) A1_3 Influencing factors of epidemic spread of infectious diseases | Experts suggested that A1_3 should be included by A1_1. | Merged to "Basic epidemiological knowledge of infectious diseases (judgment of infection source and transmission route, understanding of susceptible population, influencing factors of infectious disease epidemic, etc.)" |
| A1_2 Criteria for judging aggregates epidemics and outbreaks of infectious diseases (including nosocomial infections) A1_4 Common prevention and control measures for infectious diseases | Experts suggested that A1_2 and A1_4 should be merged. | Merged to "Criteria for judging aggregates epidemics and outbreaks of infectious diseases (including nosocomial infections) and common prevention and control measures" |
| A1_5 Clinical manifestations of common infectious diseases | Experts suggested adding "etiology knowledge" to this item, and CV did not reach the boundary value. | Revised to "Etiology knowledge and clinical knowledge of common infectious diseases" |
| A1_6 Diagnostic criteria and differential diagnosis of common infectious diseases | None of the three indicators reached the boundary values, requiring adjustment by the research team. | This item was replaced by "Laboratory testing techniques and procedures for common infectious diseases". |
| A2_3 Steps of early warning of public health emergency | CV did not reach the boundary value, requiring adjustment by the research team. | This item was retained after discussion. |
| A2_4 Theories of health emergency management | The arithmetic mean and full mark rate did not reach the boundary values, requiring adjustment by the research team. | It was retained after discussion because the mean of 4 was acceptable. |
| A2_6 Theories of risk assessment | CV did not reach the boundary value, requiring adjustment by the research team. | This item was retained after discussion. |
| A3_2 The knowledge of the prevention and control of infectious diseases and related health emergency plans in China | Experts suggested that infectious disease prevention and control and health emergency plans at provincial and municipal levels were more relevant to core public health workforce. | Revised to "The awareness of infectious disease prevention and control and health emergency plans at provincial and municipal levels". |
| A3_3 Prevention and preparedness mechanism for public health emergencies in China | The full mark rate did not reach the boundary value, requiring adjustment by the research team. | This item was retained after discussion. |
| A3_5 Response and rescue mechanism for public health emergencies in China | None of the three indicators reached the boundary values, requiring adjustment by the research team. | It was retained after discussion because the mean of 4 was acceptable. |
| B1_1 Development of emergency plans | The full mark rate did not reach the boundary value, requiring adjustment by the research team. | This item was retained after discussion. |
| B1_2 Health popularization on infectious diseases and public health emergencies | CV did not reach the boundary value, requiring adjustment by the research team. | This item was retained after discussion. |
| B1_4 Participating in emergency drills | CV did not reach the boundary value, requiring adjustment by the research team. | This item was retained after discussion. |
| B2_3 Clarifying the reporting process for public health emergencies | CV did not reach the boundary value, requiring adjustment by the research team. | This item was retained after discussion. |
| B3_4 Familiar with the methods of case management and disinfection in the field environment | CV did not reach the boundary value, requiring adjustment by the research team. | This item was retained after discussion. |
| B4_1 Ability to undertake research projects independently B4_2 Research design ability B4_3 Ability to write research papers independently B4_4 Understanding domestic and foreign status and trends in the profession | Experts pointed out that B4_1 was repeated with B4_2, B4_3, and B4_4. | B4_1 was deleted. |
| C1_3 Ability to allocate and dispatch resources needed for teamwork | CV did not reach the boundary value, requiring adjustment by the research team. | This item was retained after discussion. |
| C4_2 Regularly communicating information regarding public health needs, goals, achievements and major crises to target audience through media | Experts suggested adding a premise "permitted by the organization". | Revised to "Use the media, where permitted by the organization, to regularly communicate relevant information about public health needs, goals, achievements, and major crises to target audiences". |
| C5_1 Understanding the impact of own behaviors or reactions on team members | None of the three indicators reached the boundary values, requiring adjustment by the research team. | It was retained after discussion because the mean of 4 was acceptable. |
| C5_2 Giving proper feedback to criticisms on own behaviors or performance by others | CV did not reach the boundary value, requiring adjustment by the research team. | This item was retained after discussion. |
| C6_1 Identifying opportunities for team growth, innovation, reform and development | CV did not reach the boundary value, requiring adjustment by the research team. | This item was retained after discussion. |
| C6_2 Creating opportunities for teams to learn and improve together | The arithmetic mean and CV did not reach the boundary values, requiring adjustment by the research team. | It was retained after discussion because the mean of 4 was acceptable. |
| D1_2 The major was related to infectious disease prevention and control or health emergency | The arithmetic mean and full mark rate did not reach the boundary values, requiring adjustment by the research team. | It was retained after discussion because the mean of 4 was acceptable. |
| D1_3 Obtaining relevant professional qualification certificates | Experts pointed out that there was none relevant professional qualification certificates so far in the CDC system. | Deleted. |
| D2_1 Physical fitness | CV did not reach the boundary value, requiring adjustment by the research team. | This item was retained after discussion. |
| D2_5 Ability to continue studying and self-improvement | Experts suggested revising to "Individual willingness and ability to learn". | Revised to "Individual willingness and ability to learn". |
| D2_7 Training and guiding ability (guiding students or subordinates) | CV did not reach the boundary value, requiring adjustment by the research team. | This item was retained after discussion. |

Supplementary Table 8. Kendall’s W statistic of the first round of Delphi

| **W** | **χ²** | ***P*** |
| --- | --- | --- |
| 0.192 | 349.261 | < 0.001 |

Supplementary Table 9. Revised scale after the second round of Delphi

| **Primary items** | **Secondary items** | **Tertiary items** |
| --- | --- | --- |
|  |  |  |
|  |  |  |
| A Knowledge | A1 Knowledge of infectious diseases | A1_1 Basic epidemiological knowledge of infectious diseases (judgment of infection source and transmission route, understanding of susceptible population, influencing factors of infectious disease epidemic, etc.) |
|  |  | A1_2 Criteria for judging aggregates epidemics and outbreaks of infectious diseases (including nosocomial infections) and common prevention and control measures |
|  |  | A1_3 Etiology knowledge and clinical knowledge of common infectious diseases |
|  |  | A1_4 Laboratory testing techniques and procedures for common infectious diseases |
|  | A2 Knowledge of public health emergency management | A2_1 Fundamentals of public health emergency management |
|  |  | A2_2 Different ways of infectious disease surveillance |
|  |  | A2_3 Steps of early warning of public health emergency |
|  |  | A2_4 Theories of health emergency management |
|  |  | A2_5 Theories of crisis decision making |
|  |  | A2_6 Theories of risk assessment |
|  |  | A2_7 Theories of risk communication |
|  |  | A2_8 Command, coordination and control of health emergency |
|  | A3 Laws, plans and mechanisms for responding to public health emergencies | A3_1 The knowledge of relevant laws and regulations on infectious disease prevention and control and health emergency in China |
|  |  | A3_2 The awareness of infectious disease prevention and control and health emergency plans at provincial and municipal levels |
|  |  | A3_3 Prevention and preparedness mechanism for public health emergencies in China |
|  |  | A3_4 Surveillance and early warning mechanism for public health emergencies in China |
|  |  | A3_5 Response and rescue mechanism for public health emergencies in China |
| B Practical skills | B1 Infectious diseases prevention and emergency preparedness | B1_1 Development of emergency plans |
|  |  | B1_2 Health popularization on infectious diseases and public health emergencies |
|  |  | B1_3 Receiving professional training |
|  |  | B1_4 Participating in emergency drills |
|  |  | B1_5 Emergency capacity assessment |
|  | B2 Infectious diseases surveillance and early warning | B2_1 Clarifying the content and process of infectious diseases surveillance |
|  |  | B2_2 Ability to detect abnormal signals of public health emergencies |
|  |  | B2_3 Clarifying the reporting process for public health emergencies |
|  |  | B2_4 Determining the reliability of information sources on infectious diseases |
|  |  | B2_5 Ability to extract key information from selected information sources |
|  |  | B2_6 Predicting occurrence and epidemic trends of infectious diseases based on surveillance information |
|  | B3 Public health response to infectious diseases | B3_1 Mastering knowledge and skills of personal protection |
|  |  | B3_2 Clarifying on-site processing procedures for public health emergencies |
|  |  | B3_3 Mastering the principles of defining epidemic areas |
|  |  | B3_4 Familiar with the methods of case management and disinfection in the field environment |
|  |  | B3_5 Correctly implementing the collection, transportation and preservation of specimens from infectious disease cases |
|  |  | B3_6 Ability to correctly carry out epidemiological investigations and write investigation reports |
|  |  | B3_7 Analyzing the situation of public health incidents and proposing targeted prevention and control measures |
|  | B4 Scientific research ability | B4_1 Research design ability |
|  |  | B4_2 Ability to write research papers independently |
|  |  | B4_3 Understanding domestic and foreign status and trends in the profession |
| C Leadership | C1 Leadership fundamentals | C1_1 Proper allocation of tasks |
|  |  | C1_2 Ability to obtain resources needed for teamwork |
|  |  | C1_3 Ability to allocate and dispatch resources needed for teamwork |
|  |  | C1_4 Supervising and adjusting project implementation |
|  | C2 Decision-making ability | C2_1 Systematic understanding of current public health issues |
|  |  | C2_2 Integrating different perspectives during decision making |
|  |  | C2_3 Clarifying problems to be solved and expected outcomes of decisions |
|  |  | C2_4 Ability to formulate alternative plans and select the optimal one |
|  |  | C2_5 Ability to execute projects |
|  | C3 Team mobilization ability | C3_1 Creating an environment conducive to opinion exchange within the department |
|  |  | C3_2 Having characteristics to enable effective team leadership like integrity, enthusiasm, honesty, caring, trustworthiness, sense of responsibility, etc. |
|  |  | C3_3 Establishing effective team motivation models including listening, dialoguing, negotiating, rewarding, encouragement, inspiration, etc. |
|  | C4 Communication skills | C4_1 Ability to communicate and coordinate with superiors, subordinates and partners |
|  |  | C4_2 Use the media, where permitted by the organization, to regularly communicate relevant information about public health needs, goals, achievements, and major crises to target audiences |
|  |  | C4_3 Effectively applying negotiation skills in resolving disputes |
|  | C5 Self-regulation and interpersonal coordination abilities | C5_1 Understanding the impact of own behaviors or reactions on team members |
|  |  | C5_2 Giving proper feedback to criticisms on own behaviors or performance by others |
|  |  | C5_3 Pressure toughness and ability to deal with complex problems |
|  | C6 Team learning and development | C6_1 Identifying opportunities for team growth, innovation, reform and development |
|  |  | C6_2 Creating opportunities for teams to learn and improve together |
|  |  | C6_3 Helping members clarify thinking and turn ideas into feasible plans |
|  |  | C6_4 Post-incident learning and summarization abilities |
| D Personal quality | D1 Professional qualifications | D1_1 Having the level of education or professional training to meet the requirements of the job |
|  |  | D1_2 The major was related to infectious disease prevention and control or health emergency |
|  | D2 Professional quality | D2_1 Physical fitness |
|  |  | D2_2 Psychological quality |
|  |  | D2_3 Political literacy |
|  |  | D2_4 Abiding by the work standard and assuming the work responsibility |
|  |  | D2_5 Individual willingness and ability to learn |
|  |  | D2_6 Understanding my own work role and carry out appropriate work |
|  |  | D2_7 Training and guiding ability (guiding students or subordinates) |

Supplementary Table 10. Score comparison between high-score and low-score groups

| **Items** | **Low-score group (n=42)** | |  | **High-score group (n=43)** | | ***P*** |
| --- | --- | --- | --- | --- | --- | --- |
|  | **Mean** | **SD** |  | **Mean** | **SD** |  |
| A1_1 | 3.83 | 0.62 |  | 4.67 | 0.47 | < 0.001 |
| A1_2 | 3.40 | 0.59 |  | 4.63 | 0.54 | < 0.001 |
| A1_3 | 3.36 | 0.62 |  | 4.37 | 0.62 | < 0.001 |
| A1_4 | 3.12 | 1.02 |  | 3.93 | 0.83 | < 0.001 |
| A2_1 | 3.12 | 0.63 |  | 4.60 | 0.49 | < 0.001 |
| A2_2 | 3.17 | 0.73 |  | 4.47 | 0.59 | < 0.001 |
| A2_3 | 3.05 | 0.62 |  | 4.47 | 0.59 | < 0.001 |
| A2_4 | 2.64 | 0.66 |  | 4.26 | 0.66 | < 0.001 |
| A2_5 | 2.33 | 0.69 |  | 3.81 | 0.82 | < 0.001 |
| A2_6 | 2.74 | 0.66 |  | 4.30 | 0.56 | < 0.001 |
| A2_7 | 2.57 | 0.67 |  | 4.14 | 0.68 | < 0.001 |
| A2_8 | 2.57 | 0.67 |  | 4.28 | 0.67 | < 0.001 |
| A3_1 | 3.14 | 0.68 |  | 4.40 | 0.62 | < 0.001 |
| A3_2 | 3.05 | 0.62 |  | 4.40 | 0.54 | < 0.001 |
| A3_3 | 2.88 | 0.67 |  | 4.26 | 0.62 | < 0.001 |
| A3_4 | 2.90 | 0.73 |  | 4.37 | 0.58 | < 0.001 |
| A3_5 | 2.71 | 0.64 |  | 4.19 | 0.59 | < 0.001 |
| B1_1 | 2.14 | 0.81 |  | 3.63 | 1.13 | < 0.001 |
| B1_2 | 2.74 | 0.80 |  | 4.23 | 0.65 | < 0.001 |
| B1_3 | 2.90 | 0.79 |  | 4.19 | 0.63 | < 0.001 |
| B1_4 | 2.71 | 0.83 |  | 4.05 | 0.79 | < 0.001 |
| B1_5 | 2.86 | 0.84 |  | 4.28 | 0.63 | < 0.001 |
| B2_1 | 3.33 | 0.75 |  | 4.63 | 0.49 | < 0.001 |
| B2_2 | 3.10 | 0.73 |  | 4.49 | 0.63 | < 0.001 |
| B2_3 | 3.19 | 0.74 |  | 4.63 | 0.54 | < 0.001 |
| B2_4 | 3.07 | 0.71 |  | 4.49 | 0.63 | < 0.001 |
| B2_5 | 3.17 | 0.73 |  | 4.58 | 0.50 | < 0.001 |
| B2_6 | 2.83 | 0.58 |  | 4.47 | 0.59 | < 0.001 |
| B3_1 | 3.62 | 0.58 |  | 4.88 | 0.32 | < 0.001 |
| B3_2 | 3.33 | 0.75 |  | 4.72 | 0.55 | < 0.001 |
| B3_3 | 2.90 | 0.76 |  | 4.49 | 0.63 | < 0.001 |
| B3_4 | 3.07 | 0.71 |  | 4.51 | 0.63 | < 0.001 |
| B3_5 | 3.33 | 1.00 |  | 4.40 | 0.76 | < 0.001 |
| B3_6 | 3.19 | 0.83 |  | 4.65 | 0.61 | < 0.001 |
| B3_7 | 3.24 | 0.69 |  | 4.58 | 0.54 | < 0.001 |
| B4_1 | 2.90 | 0.73 |  | 4.02 | 0.74 | < 0.001 |
| B4_2 | 3.19 | 0.77 |  | 4.12 | 0.85 | < 0.001 |
| B4_3 | 2.69 | 0.78 |  | 3.67 | 0.71 | < 0.001 |
| C1_1 | 3.31 | 0.87 |  | 4.47 | 0.59 | < 0.001 |
| C1_2 | 3.14 | 0.72 |  | 4.35 | 0.65 | < 0.001 |
| C1_3 | 3.21 | 0.75 |  | 4.56 | 0.55 | < 0.001 |
| C1_4 | 3.26 | 0.83 |  | 4.56 | 0.55 | < 0.001 |
| C2_1 | 2.98 | 0.64 |  | 4.30 | 0.60 | < 0.001 |
| C2_2 | 2.90 | 0.66 |  | 4.30 | 0.56 | < 0.001 |
| C2_3 | 2.95 | 0.66 |  | 4.28 | 0.55 | < 0.001 |
| C2_4 | 3.00 | 0.66 |  | 4.37 | 0.62 | < 0.001 |
| C2_5 | 3.36 | 0.66 |  | 4.60 | 0.49 | < 0.001 |
| C3_1 | 3.48 | 0.63 |  | 4.60 | 0.49 | < 0.001 |
| C3_2 | 3.62 | 0.70 |  | 4.56 | 0.63 | < 0.001 |
| C3_3 | 3.52 | 0.67 |  | 4.51 | 0.59 | < 0.001 |
| C4_1 | 3.69 | 0.52 |  | 4.70 | 0.51 | < 0.001 |
| C4_2 | 3.07 | 0.64 |  | 4.37 | 0.66 | < 0.001 |
| C4_3 | 3.05 | 0.54 |  | 4.37 | 0.54 | < 0.001 |
| C5_1 | 3.55 | 0.55 |  | 4.42 | 0.63 | < 0.001 |
| C5_2 | 3.67 | 0.65 |  | 4.56 | 0.55 | < 0.001 |
| C5_3 | 3.31 | 0.64 |  | 4.56 | 0.67 | < 0.001 |
| C6_1 | 3.38 | 0.62 |  | 4.49 | 0.59 | < 0.001 |
| C6_2 | 3.29 | 0.67 |  | 4.47 | 0.55 | < 0.001 |
| C6_3 | 3.36 | 0.66 |  | 4.42 | 0.63 | < 0.001 |
| C6_4 | 3.62 | 0.62 |  | 4.65 | 0.48 | < 0.001 |
| D1_1 | 3.64 | 0.62 |  | 4.79 | 0.41 | < 0.001 |
| D1_2 | 3.36 | 0.88 |  | 4.67 | 0.57 | < 0.001 |
| D2_1 | 3.67 | 0.72 |  | 4.74 | 0.54 | < 0.001 |
| D2_2 | 3.79 | 0.72 |  | 4.84 | 0.43 | < 0.001 |
| D2_3 | 4.02 | 0.52 |  | 4.88 | 0.39 | < 0.001 |
| D2_4 | 4.33 | 0.48 |  | 4.91 | 0.29 | < 0.001 |
| D2_5 | 4.10 | 0.48 |  | 4.81 | 0.39 | < 0.001 |
| D2_6 | 4.02 | 0.52 |  | 4.88 | 0.32 | < 0.001 |
| D2_7 | 3.57 | 0.77 |  | 4.63 | 0.58 | < 0.001 |

Supplementary Table 11. Results of Cronbach's Alpha of the scale based on the pilot survey and the formal survey

| Primary items | Cronbach's Alpha | |
| --- | --- | --- |
|  | Pilot survey | Formal survey |
| A Knowledge | 0.954 | 0.942 |
| B Practical skills | 0.949 | 0.954 |
| C Leadership | 0.965 | 0.972 |
| D Personal quality | 0.893 | 0.898 |

Supplementary Table 12. Inspection results of scale items

| **Items** | **Cronbach’s alpha of item-total statistics** | | | |  | **Item-total correlation test** | |  |
| --- | --- | --- | --- | --- | --- | --- | --- | --- |
|  | **Scale mean if item deleted** | **Scale variance if item deleted** | **Corrected item-total correlation** | **Cronbach’s Alpha if item deleted** |  | **r** | ***P*** |  |
| A1_1 | 259.10 | 1257.14 | 0.57 | 0.981 |  | 0.578 | < 0.001 |  |
| A1_2 | 259.38 | 1247.64 | 0.67 | 0.981 |  | 0.679 | < 0.001 |  |
| A1_3 | 259.55 | 1253.56 | 0.56 | 0.981 |  | 0.571 | < 0.001 |  |
| A1_4 | 259.89 | 1258.14 | 0.33 | 0.981 |  | 0.350 | < 0.001 |  |
| A2_1 | 259.55 | 1241.66 | 0.73 | 0.981 |  | 0.744 | < 0.001 |  |
| A2_2 | 259.59 | 1243.91 | 0.69 | 0.981 |  | 0.703 | < 0.001 |  |
| A2_3 | 259.70 | 1241.00 | 0.75 | 0.981 |  | 0.757 | < 0.001 |  |
| A2_4 | 259.97 | 1238.16 | 0.70 | 0.981 |  | 0.708 | < 0.001 |  |
| A2_5 | 260.37 | 1241.74 | 0.62 | 0.981 |  | 0.632 | < 0.001 |  |
| A2_6 | 259.91 | 1237.34 | 0.72 | 0.981 |  | 0.731 | < 0.001 |  |
| A2_7 | 260.06 | 1237.55 | 0.71 | 0.981 |  | 0.725 | < 0.001 |  |
| A2_8 | 259.99 | 1232.72 | 0.75 | 0.981 |  | 0.759 | < 0.001 |  |
| A3_1 | 259.62 | 1244.63 | 0.71 | 0.981 |  | 0.716 | < 0.001 |  |
| A3_2 | 259.65 | 1243.11 | 0.75 | 0.981 |  | 0.758 | < 0.001 |  |
| A3_3 | 259.85 | 1241.52 | 0.72 | 0.981 |  | 0.729 | < 0.001 |  |
| A3_4 | 259.81 | 1238.42 | 0.76 | 0.981 |  | 0.766 | < 0.001 |  |
| A3_5 | 259.98 | 1239.43 | 0.76 | 0.981 |  | 0.765 | < 0.001 |  |
| B1_1 | 260.45 | 1241.52 | 0.53 | 0.981 |  | 0.550 | < 0.001 |  |
| B1_2 | 259.88 | 1240.49 | 0.65 | 0.981 |  | 0.666 | < 0.001 |  |
| B1_3 | 259.87 | 1246.19 | 0.61 | 0.981 |  | 0.628 | < 0.001 |  |
| B1_4 | 260.03 | 1245.65 | 0.56 | 0.981 |  | 0.580 | < 0.001 |  |
| B1_5 | 259.94 | 1241.28 | 0.62 | 0.981 |  | 0.639 | < 0.001 |  |
| B2_1 | 259.43 | 1243.70 | 0.70 | 0.981 |  | 0.709 | < 0.001 |  |
| B2_2 | 259.61 | 1242.85 | 0.68 | 0.981 |  | 0.688 | < 0.001 |  |
| B2_3 | 259.51 | 1243.11 | 0.67 | 0.981 |  | 0.680 | < 0.001 |  |
| B2_4 | 259.61 | 1241.16 | 0.70 | 0.981 |  | 0.714 | < 0.001 |  |
| B2_5 | 259.47 | 1240.78 | 0.73 | 0.981 |  | 0.744 | < 0.001 |  |
| B2_6 | 259.72 | 1236.24 | 0.77 | 0.980 |  | 0.781 | < 0.001 |  |
| B3_1 | 259.17 | 1247.26 | 0.75 | 0.981 |  | 0.756 | < 0.001 |  |
| B3_2 | 259.44 | 1239.69 | 0.76 | 0.981 |  | 0.766 | < 0.001 |  |
| B3_3 | 259.70 | 1236.59 | 0.73 | 0.981 |  | 0.741 | < 0.001 |  |
| B3_4 | 259.65 | 1240.04 | 0.69 | 0.981 |  | 0.705 | < 0.001 |  |
| B3_5 | 259.63 | 1249.79 | 0.47 | 0.981 |  | 0.493 | < 0.001 |  |
| B3_6 | 259.47 | 1239.07 | 0.67 | 0.981 |  | 0.680 | < 0.001 |  |
| B3_7 | 259.50 | 1239.86 | 0.73 | 0.981 |  | 0.740 | < 0.001 |  |
| B4_1 | 259.98 | 1248.24 | 0.57 | 0.981 |  | 0.589 | < 0.001 |  |
| B4_2 | 259.81 | 1251.21 | 0.52 | 0.981 |  | 0.537 | < 0.001 |  |
| B4_3 | 260.28 | 1249.27 | 0.58 | 0.981 |  | 0.593 | < 0.001 |  |
| C1_1 | 259.54 | 1243.94 | 0.71 | 0.981 |  | 0.721 | < 0.001 |  |
| C1_2 | 259.70 | 1244.99 | 0.66 | 0.981 |  | 0.670 | < 0.001 |  |
| C1_3 | 259.60 | 1241.12 | 0.72 | 0.981 |  | 0.733 | < 0.001 |  |
| C1_4 | 259.60 | 1240.83 | 0.69 | 0.981 |  | 0.705 | < 0.001 |  |
| C2_1 | 259.79 | 1241.36 | 0.75 | 0.981 |  | 0.763 | < 0.001 |  |
| C2_2 | 259.75 | 1239.90 | 0.77 | 0.981 |  | 0.779 | < 0.001 |  |
| C2_3 | 259.77 | 1240.75 | 0.78 | 0.980 |  | 0.785 | < 0.001 |  |
| C2_4 | 259.68 | 1240.17 | 0.76 | 0.981 |  | 0.768 | < 0.001 |  |
| C2_5 | 259.43 | 1244.78 | 0.73 | 0.981 |  | 0.742 | < 0.001 |  |
| C3_1 | 259.39 | 1250.08 | 0.63 | 0.981 |  | 0.644 | < 0.001 |  |
| C3_2 | 259.37 | 1253.47 | 0.56 | 0.981 |  | 0.575 | < 0.001 |  |
| C3_3 | 259.43 | 1252.13 | 0.59 | 0.981 |  | 0.603 | < 0.001 |  |
| C4_1 | 259.28 | 1253.88 | 0.66 | 0.981 |  | 0.672 | < 0.001 |  |
| C4_2 | 259.77 | 1245.19 | 0.63 | 0.981 |  | 0.643 | < 0.001 |  |
| C4_3 | 259.74 | 1244.65 | 0.71 | 0.981 |  | 0.721 | < 0.001 |  |
| C5_1 | 259.47 | 1255.39 | 0.60 | 0.981 |  | 0.609 | < 0.001 |  |
| C5_2 | 259.37 | 1257.16 | 0.57 | 0.981 |  | 0.579 | < 0.001 |  |
| C5_3 | 259.48 | 1247.16 | 0.67 | 0.981 |  | 0.686 | < 0.001 |  |
| C6_1 | 259.55 | 1248.74 | 0.66 | 0.981 |  | 0.673 | < 0.001 |  |
| C6_2 | 259.61 | 1247.43 | 0.65 | 0.981 |  | 0.666 | < 0.001 |  |
| C6_3 | 259.51 | 1250.17 | 0.66 | 0.981 |  | 0.668 | < 0.001 |  |
| C6_4 | 259.33 | 1251.11 | 0.67 | 0.981 |  | 0.677 | < 0.001 |  |
| D1_1 | 259.23 | 1250.14 | 0.67 | 0.981 |  | 0.683 | < 0.001 |  |
| D1_2 | 259.37 | 1247.29 | 0.53 | 0.981 |  | 0.552 | < 0.001 |  |
| D2_1 | 259.23 | 1252.93 | 0.56 | 0.981 |  | 0.570 | < 0.001 |  |
| D2_2 | 259.18 | 1251.02 | 0.62 | 0.981 |  | 0.636 | < 0.001 |  |
| D2_3 | 258.97 | 1258.44 | 0.58 | 0.981 |  | 0.586 | < 0.001 |  |
| D2_4 | 258.87 | 1265.87 | 0.46 | 0.981 |  | 0.474 | < 0.001 |  |
| D2_5 | 259.05 | 1262.24 | 0.51 | 0.981 |  | 0.525 | < 0.001 |  |
| D2_6 | 259.03 | 1259.20 | 0.58 | 0.981 |  | 0.588 | < 0.001 |  |
| D2_7 | 259.29 | 1251.65 | 0.58 | 0.981 |  | 0.590 | < 0.001 |  |

Supplementary Table 13. Factor loadings of items in the knowledge section (first round of exploratory factor analysis)

| Items | Factor 1 | Factor 2 | Factor 3 |
| --- | --- | --- | --- |
| A3_3 | **0.833** | 0.371 | 0.172 |
| A3_4 | **0.829** | 0.379 | 0.220 |
| A3_1 | **0.805** | 0.223 | 0.346 |
| A3_2 | **0.792** | 0.364 | 0.167 |
| A3_5 | **0.682** | 0.523 | 0.238 |
| A2_3 | **0.612** | 0.507 | 0.286 |
| A2_2 | **0.585** | 0.376 | 0.381 |
| A2_5 | 0.300 | **0.827** | 0.106 |
| A2_7 | 0.281 | **0.815** | 0.290 |
| A2_8 | 0.355 | **0.771** | 0.245 |
| A2_6 | 0.375 | **0.736** | 0.279 |
| A2_4 | 0.508 | **0.673** | 0.188 |
| A2_1 | 0.473 | **0.609** | 0.400 |
| A1_1 | 0.109 | 0.176 | **0.883** |
| A1_3 | 0.284 | 0.189 | **0.765** |
| A1_2 | 0.301 | 0.282 | **0.749** |

Supplementary Table 14. Factor loadings of items in the section of practical skills (first round of exploratory factor analysis)

| Items | Factor 1 | Factor 2 | Factor 3 | Factor 4 |
| --- | --- | --- | --- | --- |
| B2_2 | **0.835** | 0.187 | 0.234 | 0.153 |
| B2_3 | **0.786** | 0.285 | 0.259 |  |
| B2_4 | **0.775** | 0.301 | 0.234 | 0.174 |
| B2_1 | **0.758** | 0.273 | 0.255 | 0.247 |
| B2_5 | **0.756** | 0.378 | 0.237 | 0.150 |
| B2_6 | **0.674** | 0.412 | 0.354 | 0.203 |
| B3_1 | **0.487** | 0.413 | 0.301 | 0.336 |
| B3_4 | 0.224 | **0.719** | 0.299 | 0.192 |
| B3_6 | 0.500 | **0.715** |  | 0.138 |
| B3_7 | 0.489 | **0.673** | 0.133 | 0.284 |
| B3_3 | 0.453 | **0.671** | 0.266 | 0.123 |
| B3_2 | 0.498 | **0.592** | 0.303 | 0.200 |
| B1_4 | 0.202 |  | **0.839** | 0.120 |
| B1_5 | 0.224 | 0.254 | **0.740** | 0.112 |
| B1_3 | 0.348 |  | **0.706** | 0.231 |
| B1_1 | 0.134 | 0.458 | **0.565** |  |
| B1_2 | 0.386 | 0.392 | **0.563** |  |
| B4_1 | 0.178 |  | 0.165 | **0.889** |
| B4_2 | 0.181 | 0.115 |  | **0.888** |
| B4_3 | 0.125 | 0.239 | 0.131 | **0.797** |

Supplementary Table 15. Factor loadings of items in the leadership section (first round of exploratory factor analysis)

| Items | Factor 1 | Factor 2 | Factor 3 | Factor 4 | Factor 5 | Factor 6 |
| --- | --- | --- | --- | --- | --- | --- |
| C2_1 | **0.789** | 0.152 | 0.220 | 0.174 | 0.199 | 0.189 |
| C2_2 | **0.771** | 0.245 | 0.295 | 0.190 | 0.256 |  |
| C2_3 | **0.768** | 0.236 | 0.303 | 0.218 | 0.183 | 0.186 |
| C2_4 | **0.748** | 0.250 | 0.305 | 0.151 | 0.230 | 0.179 |
| C2_5 | **0.650** | 0.307 | 0.319 | 0.290 | 0.168 |  |
| C5_2 | 0.104 | **0.771** | 0.227 | 0.120 | 0.181 | 0.191 |
| C5_1 | 0.239 | **0.740** | 0.156 | 0.118 | 0.303 |  |
| C5_3 | 0.368 | **0.717** |  | 0.170 | 0.191 | 0.227 |
| C4_1 | 0.208 | **0.599** | 0.264 | 0.334 |  | 0.240 |
| C1_3 | 0.315 | 0.236 | **0.764** | 0.328 | 0.177 | 0.172 |
| C1_4 | 0.336 | 0.253 | **0.760** | 0.278 | 0.155 | 0.143 |
| C1_2 | 0.311 | 0.145 | **0.737** | 0.181 | 0.265 | 0.247 |
| C1_1 | 0.406 | 0.250 | **0.702** | 0.274 | 0.167 |  |
| C3_2 | 0.189 | 0.146 | 0.201 | **0.849** | 0.242 | 0.104 |
| C3_3 | 0.187 | 0.168 | 0.278 | **0.826** | 0.254 |  |
| C3_1 | 0.301 | 0.245 | 0.276 | **0.713** |  | 0.218 |
| C6_3 | 0.338 | 0.308 | 0.234 | 0.259 | **0.717** |  |
| C6_2 | 0.223 | 0.235 | 0.248 | 0.248 | **0.715** | 0.332 |
| C6_1 | 0.343 | 0.373 | 0.151 | 0.271 | **0.600** | 0.266 |
| C6_4 | 0.347 | 0.523 | 0.276 | 0.168 | **0.524** |  |
| C4_2 | 0.219 | 0.299 | 0.160 | 0.202 | 0.163 | **0.798** |
| C4_3 | 0.337 | 0.468 | 0.273 | 0.152 | 0.229 | **0.541** |

Supplementary Table 16. Factor loadings of items in the section of personal quality

| Items | Factor 1 | Factor 2 |
| --- | --- | --- |
| D2_5 | **0.828** | 0.126 |
| D2_2 | **0.823** | 0.205 |
| D2_6 | **0.798** | 0.255 |
| D2_3 | **0.783** | 0.241 |
| D2_4 | **0.757** | 0.220 |
| D2_1 | **0.689** | 0.302 |
| D2_7 | **0.636** | 0.415 |
| D1_2 | 0.123 | **0.921** |
| D1_1 | 0.410 | **0.767** |

Supplementary Table 17. Factor loadings of items in the knowledge section (second round of exploratory factor analysis)

| Items | Factor 1 | Factor 2 | Factor 3 |
| --- | --- | --- | --- |
| A2_5 | **0.826** | 0.295 | 0.123 |
| A2_7 | **0.808** | 0.286 | 0.296 |
| A2_8 | **0.775** | 0.339 | 0.271 |
| A2_6 | **0.748** | 0.332 | 0.300 |
| A2_4 | **0.684** | 0.473 | 0.195 |
| A2_1 | **0.617** | 0.439 | 0.402 |
| A3_3 | 0.382 | **0.845** | 0.196 |
| A3_4 | 0.391 | **0.824** | 0.244 |
| A3_1 | 0.236 | **0.794** | 0.351 |
| A3_2 | 0.380 | **0.778** | 0.217 |
| A3_5 | 0.526 | **0.690** | 0.267 |
| A1_1 | 0.160 | 0.101 | **0.883** |
| A1_2 | 0.277 | 0.274 | **0.762** |
| A1_3 | 0.176 | 0.285 | **0.757** |
| B3_1 | 0.41128 | 0.2587 | 0.55392 |

Supplementary Table 18. Factor loadings of items in the section of practical skills (second round of exploratory factor analysis)

| Items | Factor 1 | Factor 2 | Factor 3 | Factor 4 |
| --- | --- | --- | --- | --- |
| B2_2 | **0.836** | 0.188 | 0.236 | 0.156 |
| B2_3 | **0.786** | 0.286 | 0.260 |  |
| B2_4 | **0.776** | 0.301 | 0.236 | 0.177 |
| B2_1 | **0.758** | 0.270 | 0.258 | 0.246 |
| B2_5 | **0.757** | 0.375 | 0.239 | 0.149 |
| B2_6 | **0.677** | 0.414 | 0.356 | 0.208 |
| B3_4 | 0.226 | **0.717** | 0.298 | 0.192 |
| B3_6 | 0.503 | **0.713** |  | 0.140 |
| B3_3 | 0.456 | **0.674** | 0.265 | 0.128 |
| B3_7 | 0.491 | **0.671** | 0.133 | 0.285 |
| B3_2 | 0.497 | **0.587** | 0.304 | 0.195 |
| B1_4 | 0.200 |  | **0.840** | 0.118 |
| B1_5 | 0.222 | 0.257 | **0.740** | 0.111 |
| B1_3 | 0.346 |  | **0.708** | 0.226 |
| B1_2 | 0.384 | 0.390 | **0.563** |  |
| B1_1 | 0.134 | 0.469 | **0.562** |  |
| B4_1 | 0.178 |  | 0.168 | **0.890** |
| B4_2 | 0.181 | 0.114 |  | **0.888** |
| B4_3 | 0.126 | 0.239 | 0.132 | **0.799** |

Supplementary Table 19. Factor loadings of items in the leadership section (second round of exploratory factor analysis)

| Items | Factor 1 | Factor 2 | Factor 3 | Factor 4 | Factor 5 | Factor 6 |
| --- | --- | --- | --- | --- | --- | --- |
| C2_1 | **0.788** | 0.223 | 0.148 | 0.174 | 0.197 | 0.190 |
| C2_2 | **0.772** | 0.300 | 0.234 | 0.187 | 0.262 |  |
| C2_3 | **0.770** | 0.307 | 0.209 | 0.212 | 0.201 | 0.192 |
| C2_4 | **0.747** | 0.308 | 0.254 | 0.157 | 0.214 | 0.181 |
| C2_5 | **0.650** | 0.324 | 0.306 | 0.297 | 0.152 |  |
| C1_3 | 0.312 | **0.768** | 0.224 | 0.331 | 0.172 | 0.182 |
| C1_4 | 0.331 | **0.764** | 0.261 | 0.290 | 0.125 | 0.153 |
| C1_2 | 0.313 | **0.738** | 0.132 | 0.180 | 0.265 | 0.241 |
| C1_1 | 0.411 | **0.707** | 0.207 | 0.262 | 0.197 |  |
| C5_1 | 0.233 | 0.165 | **0.784** | 0.150 | 0.230 | 0.108 |
| C5_2 | 0.100 | 0.239 | **0.754** | 0.131 | 0.171 | 0.234 |
| C5_3 | 0.367 |  | **0.707** | 0.184 | 0.174 | 0.261 |
| C6_4 | 0.345 | 0.282 | **0.558** | 0.183 | 0.480 |  |
| C3_2 | 0.190 | 0.204 | 0.135 | **0.852** | 0.241 | 0.107 |
| C3_3 | 0.189 | 0.281 | 0.148 | **0.824** | 0.262 |  |
| C3_1 | 0.297 | 0.281 | 0.237 | **0.724** |  | 0.235 |
| C6_2 | 0.226 | 0.251 | 0.229 | 0.240 | **0.730** | 0.319 |
| C6_3 | 0.339 | 0.238 | 0.332 | 0.260 | **0.702** |  |
| C6_1 | 0.346 | 0.157 | 0.351 | 0.260 | **0.625** | 0.267 |
| C4_2 | 0.219 | 0.164 | 0.266 | 0.205 | 0.175 | **0.808** |
| C4_3 | 0.338 | 0.279 | 0.453 | 0.161 | 0.217 | **0.554** |

Supplementary Table 20. Factor loadings of items in the leadership section (third round of exploratory factor analysis)

| Items | Factor 1 | Factor 2 | Factor 3 | Factor 4 | Factor 5 |
| --- | --- | --- | --- | --- | --- |
| C2_1 | **0.792** | 0.221 | 0.229 | 0.180 | 0.186 |
| C2_3 | **0.764** | 0.289 | 0.319 | 0.216 | 0.180 |
| C2_2 | **0.759** | 0.240 | 0.321 | 0.173 | 0.279 |
| C2_4 | **0.754** | 0.304 | 0.313 | 0.160 | 0.199 |
| C2_5 | **0.635** | 0.293 | 0.346 | 0.272 | 0.184 |
| C5_2 |  | **0.734** | 0.274 |  | 0.305 |
| C5_3 | 0.342 | **0.716** | 0.120 | 0.132 | 0.267 |
| C4_2 | 0.299 | **0.695** |  | 0.312 |  |
| C4_3 | 0.379 | **0.693** | 0.246 | 0.207 | 0.124 |
| C5_1 | 0.214 | **0.665** | 0.195 |  | 0.366 |
| C4_1 | 0.174 | **0.615** | 0.304 | 0.291 | 0.156 |
| C1_3 | 0.314 | 0.284 | **0.768** | 0.328 | 0.164 |
| C1_4 | 0.339 | 0.290 | **0.761** | 0.286 | 0.112 |
| C1_1 | 0.374 | 0.177 | **0.736** | 0.232 | 0.231 |
| C1_2 | 0.338 | 0.256 | **0.716** | 0.215 | 0.188 |
| C3_2 | 0.188 | 0.174 | 0.215 | **0.850** | 0.229 |
| C3_3 | 0.176 | 0.173 | 0.300 | **0.812** | 0.272 |
| C3_1 | 0.295 | 0.317 | 0.291 | **0.710** |  |
| C6_3 | 0.343 | 0.271 | 0.250 | 0.250 | **0.735** |
| C6_2 | 0.267 | 0.372 | 0.226 | 0.292 | **0.649** |
| C6_1 | 0.360 | 0.449 | 0.155 | 0.284 | **0.587** |

Supplementary Table 21. The revision methods of items not passing the item analysis or EFA

| Original items | Reasons for revision | Revision methods |
| --- | --- | --- |
| A1_4 Laboratory testing techniques and procedures for common infectious diseases | The correlation coefficient between the item score and total score was less than 0.4. | This item was deleted because it was included in "A1_3 Etiology knowledge and clinical knowledge of common infectious diseases". |
| A2_2 Different ways of infectious disease surveillance | This item was not classified under the secondary item "A2 Knowledge of public health emergency management" in the exploratory factor analysis. | This item was deleted because it was included in "B2 Infectious diseases surveillance and early warning". |
| A2_3 Steps of early warning of public health emergency | This item was not classified under the secondary item "A2 Knowledge of public health emergency management" in the exploratory factor analysis. | This item was deleted because it was included in "B2 Infectious diseases surveillance and early warning". |
| B3_1 Mastering knowledge and skills of personal protection | In exploratory factor analysis, this item had a factor loading of less than 0.5. | This item was retained after group discussion, but it was moved to be under the secondary item "A1 Knowledge of infectious diseases". |
| B3_5 Correctly implementing the collection, transportation and preservation of specimens from infectious disease cases | The correlation coefficient between the item score and total score was less than 0.4. | This item was merged with B3_4 into a new item "Familiar with the methods of case management, specimen collection, and disinfection in the field environment". |
| C4_1 Ability to communicate and coordinate with superiors, subordinates and partners | This item was not classified under the secondary item "C4 Communication skills" in the exploratory factor analysis. | This problem was solved after we merged C4 and C5 to be a new secondary item "Self-regulation and communication abilities". |
| C6_4 Post-incident learning and summarization abilities | This item was not classified under the secondary item "C6 Team learning and development" in the exploratory factor analysis. | This item was deleted because the validity test could not be satisfied by adjusting this item to other secondary indicators. |

Supplementary Table 22. Revised scale after item analysis and exploratory factor analysis

| **Primary items** | **Secondary items** | **Tertiary items** |
| --- | --- | --- |
|  |  |  |
|  |  |  |
| A Knowledge | A1 Knowledge of infectious diseases | A1_1 Basic epidemiological knowledge of infectious diseases (judgment of infection source and transmission route, understanding of susceptible population, influencing factors of infectious disease epidemic, etc.) |
|  |  | A1_2 Criteria for judging aggregates epidemics and outbreaks of infectious diseases (including nosocomial infections) and common prevention and control measures |
|  |  | A1_3 Etiology knowledge and clinical knowledge of common infectious diseases |
|  |  | A1_4 Mastering knowledge and skills of personal protection |
|  | A2 Knowledge of public health emergency management | A2_1 Fundamentals of public health emergency management |
|  |  | A2_2 Theories of health emergency management |
|  |  | A2_3 Theories of crisis decision making |
|  |  | A2_4 Theories of risk assessment |
|  |  | A2_5 Theories of risk communication |
|  |  | A2_6 Command, coordination and control of health emergency |
|  | A3 Laws, plans and mechanisms for responding to public health emergencies | A3_1 The knowledge of relevant laws and regulations on infectious disease prevention and control and health emergency in China |
|  |  | A3_2 The awareness of infectious disease prevention and control and health emergency plans at provincial and municipal levels |
|  |  | A3_3 Prevention and preparedness mechanism for public health emergencies in China |
|  |  | A3_4 Surveillance and early warning mechanism for public health emergencies in China |
|  |  | A3_5 Response and rescue mechanism for public health emergencies in China |
| B Practical skills | B1 Infectious diseases prevention and emergency preparedness | B1_1 Development of emergency plans |
|  |  | B1_2 Health popularization on infectious diseases and public health emergencies |
|  |  | B1_3 Receiving professional training |
|  |  | B1_4 Participating in emergency drills |
|  |  | B1_5 Emergency capacity assessment |
|  | B2 Infectious diseases surveillance and early warning | B2_1 Clarifying the content and process of infectious diseases surveillance |
|  |  | B2_2 Ability to detect abnormal signals of public health emergencies |
|  |  | B2_3 Clarifying the reporting process for public health emergencies |
|  |  | B2_4 Determining the reliability of information sources on infectious diseases |
|  |  | B2_5 Ability to extract key information from selected information sources |
|  |  | B2_6 Predicting occurrence and epidemic trends of infectious diseases based on surveillance information |
|  | B3 Public health response to infectious diseases | B3_1 Clarifying on-site processing procedures for public health emergencies |
|  |  | B3_2 Mastering the principles of defining epidemic areas |
|  |  | B3_3 Familiar with the methods of case management, specimen collection, and disinfection in the field environment |
|  |  | B3_4 Ability to correctly carry out epidemiological investigations and write investigation reports |
|  |  | B3_5 Analyzing the situation of public health incidents and proposing targeted prevention and control measures |
|  | B4 Scientific research ability | B4_1 Research design ability |
|  |  | B4_2 Ability to write research papers independently |
|  |  | B4_3 Understanding domestic and foreign status and trends in the profession |
| C Leadership | C1 Leadership fundamentals | C1_1 Proper allocation of tasks |
|  |  | C1_2 Ability to obtain resources needed for teamwork |
|  |  | C1_3 Ability to allocate and dispatch resources needed for teamwork |
|  |  | C1_4 Supervising and adjusting project implementation |
|  | C2 Decision-making ability | C2_1 Systematic understanding of current public health issues |
|  |  | C2_2 Integrating different perspectives during decision making |
|  |  | C2_3 Clarifying problems to be solved and expected outcomes of decisions |
|  |  | C2_4 Ability to formulate alternative plans and select the optimal one |
|  |  | C2_5 Ability to execute projects |
|  | C3 Team mobilization ability | C3_1 Creating an environment conducive to opinion exchange within the department |
|  |  | C3_2 Having characteristics to enable effective team leadership like integrity, enthusiasm, honesty, caring, trustworthiness, sense of responsibility, etc. |
|  |  | C3_3 Establishing effective team motivation models including listening, dialoguing, negotiating, rewarding, encouragement, inspiration, etc. |
|  | C4 Self-regulation and communication abilities | C4_1 Ability to communicate and coordinate with superiors, subordinates and partners |
|  |  | C4_2 Use the media, where permitted by the organization, to regularly communicate relevant information about public health needs, goals, achievements, and major crises to target audiences |
|  |  | C4_3 Effectively applying negotiation skills in resolving disputes |
|  |  | C4_4 Understanding the impact of own behaviors or reactions on team members |
|  |  | C4_5 Giving proper feedback to criticisms on own behaviors or performance by others |
|  |  | C4_6 Pressure toughness and ability to deal with complex problems |
|  | C5 Team learning and development | C5_1 Identifying opportunities for team growth, innovation, reform and development |
|  |  | C5_2 Creating opportunities for teams to learn and improve together |
|  |  | C5_3 Helping members clarify thinking and turn ideas into feasible plans |
| D Personal quality | D1 Professional qualifications | D1_1 Having the level of education or professional training to meet the requirements of the job |
|  |  | D1_2 The major was related to infectious disease prevention and control or health emergency |
|  | D2 Professional quality | D2_1 Physical fitness |
|  |  | D2_2 Psychological quality |
|  |  | D2_3 Political literacy |
|  |  | D2_4 Abiding by the work standard and assuming the work responsibility |
|  |  | D2_5 Individual willingness and ability to learn |
|  |  | D2_6 Understanding my own work role and carry out appropriate work |
|  |  | D2_7 Training and guiding ability (guiding students or subordinates) |


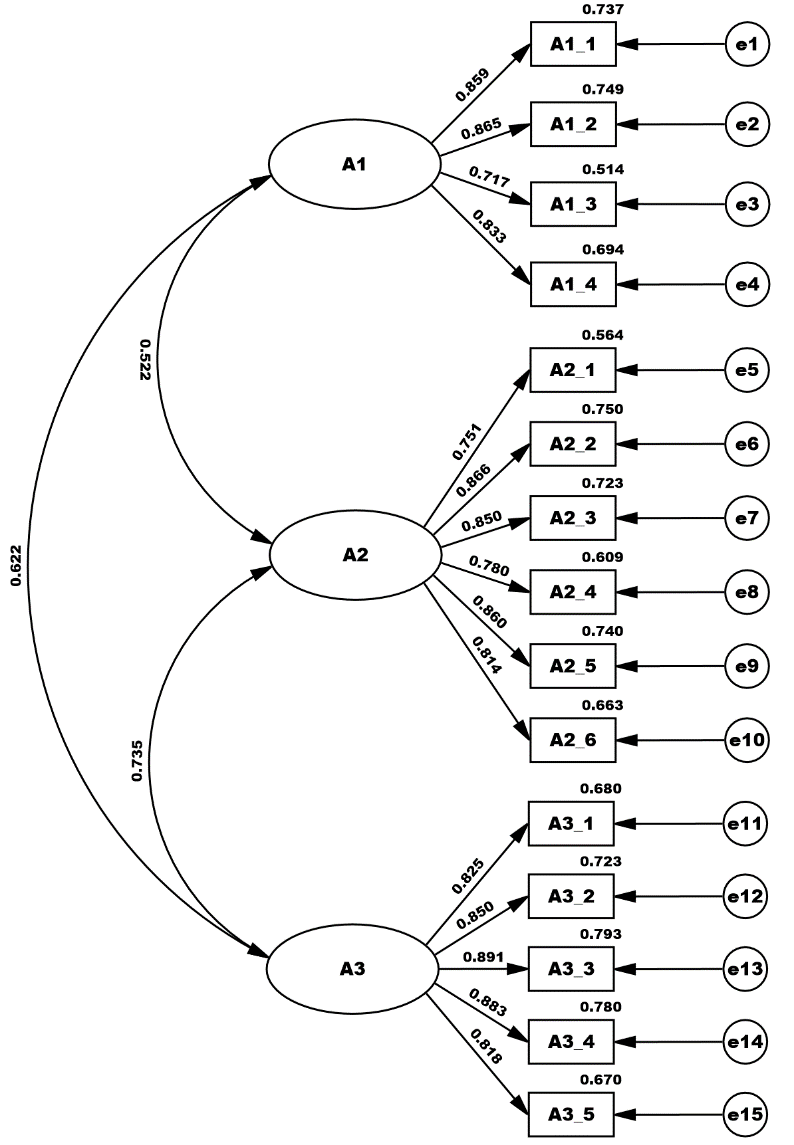


Supplementary Figure 1. Path diagram for knowledge section derived by confirmatory factor analysis.

Note: Maximum likelihood estimation. Standardized estimates for associations and residuals (e). N=476.


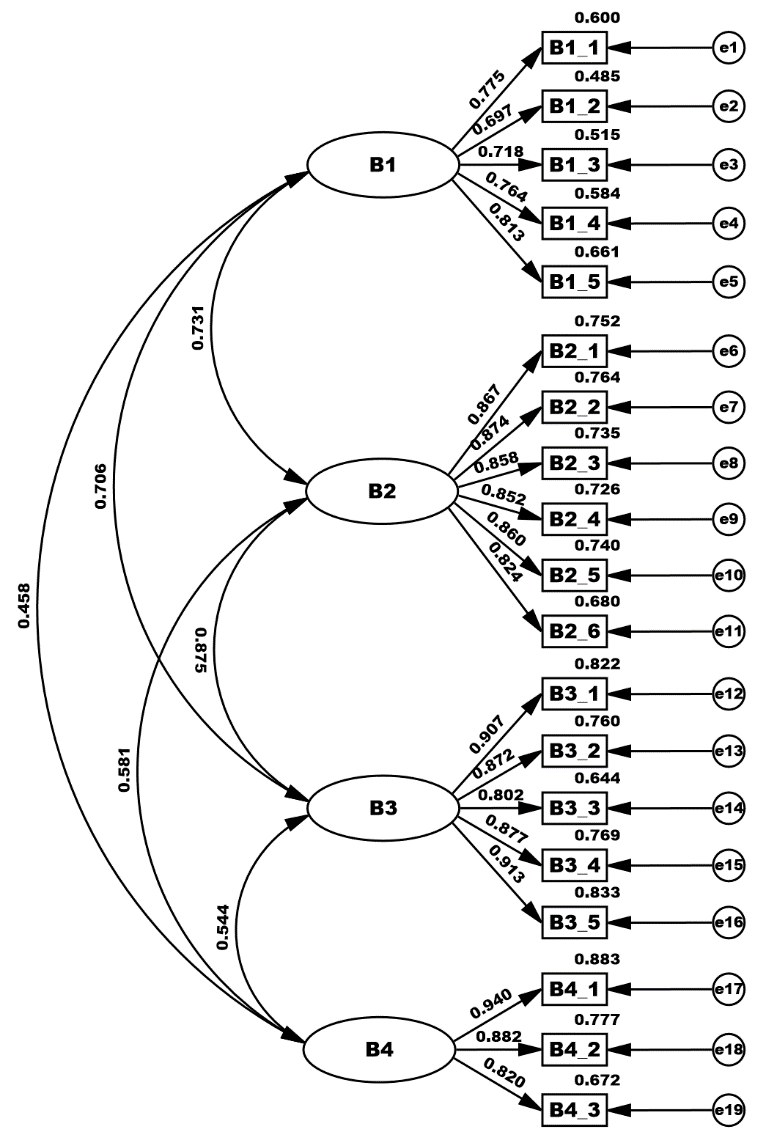


Supplementary Figure 2. Path diagram for the section of practical skills derived by confirmatory factor analysis.

Note: Maximum likelihood estimation. Standardized estimates for associations and residuals (e). N=476.


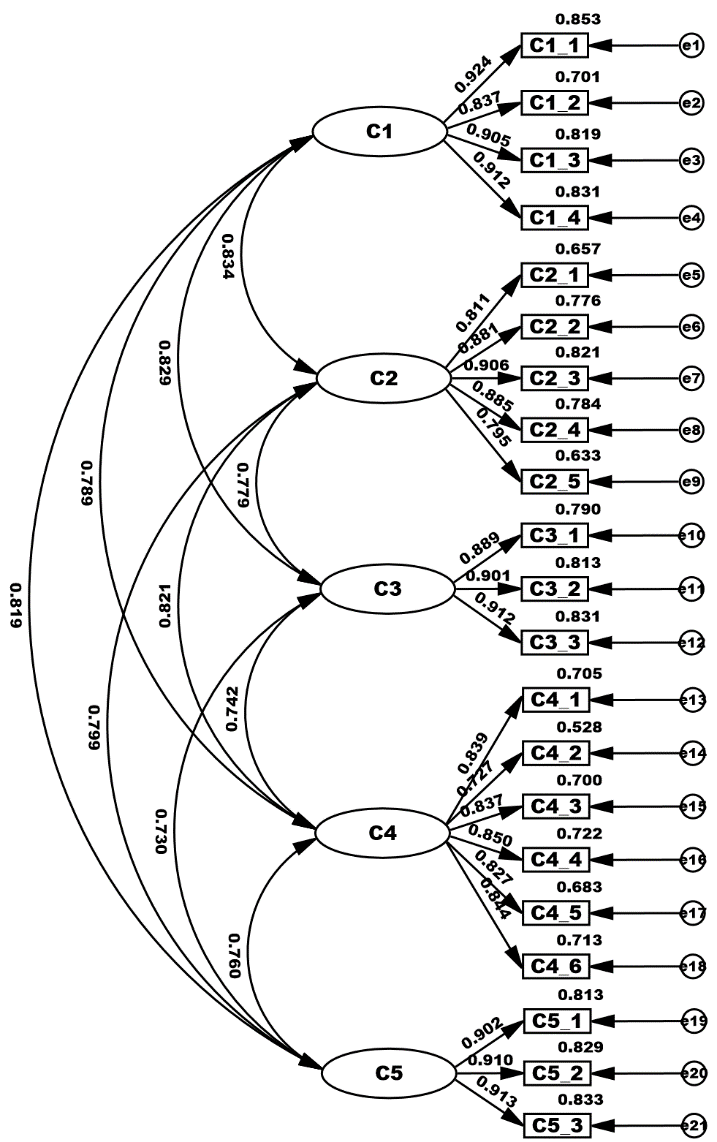


Supplementary Figure 3. Path diagram for the leadership section derived by confirmatory factor analysis.

Note: Maximum likelihood estimation. Standardized estimates for associations and residuals (e). N=476.


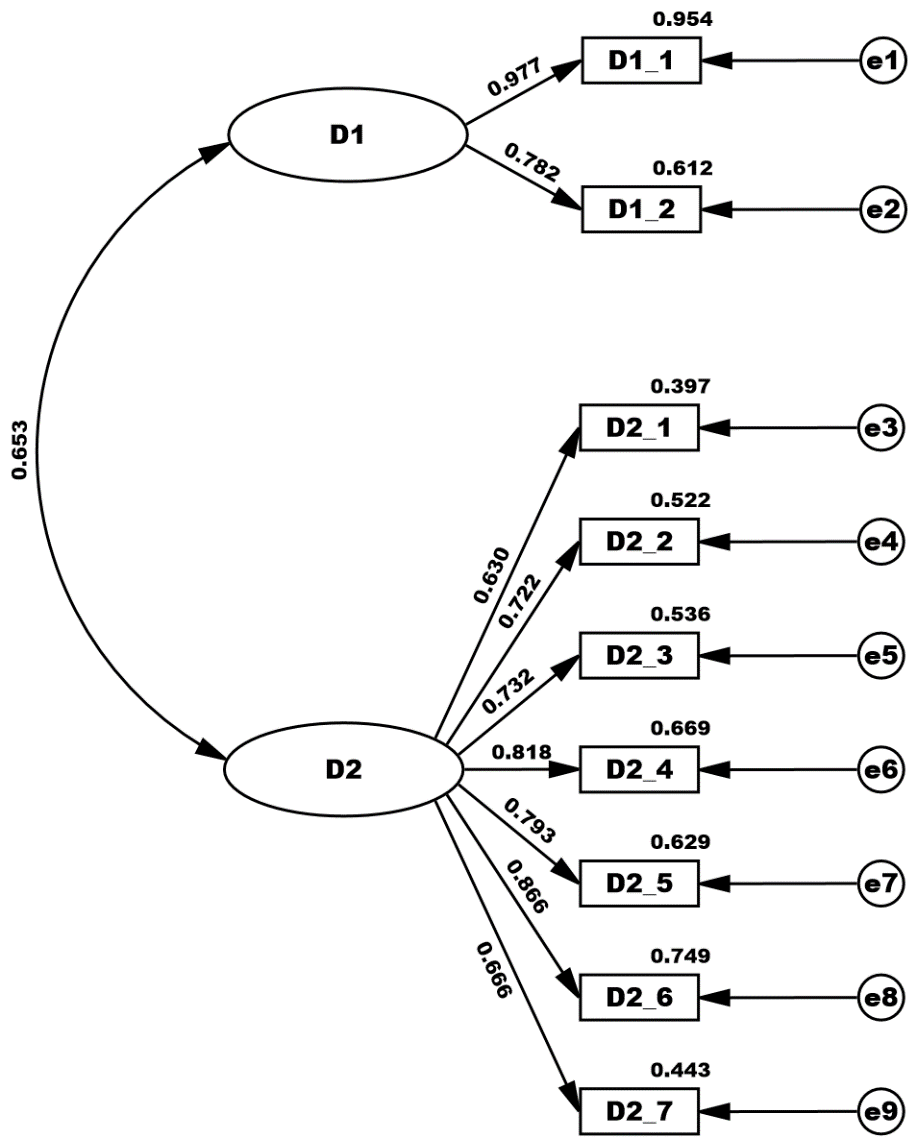


Supplementary Figure 4. Path diagram for the section of personal quality derived by confirmatory factor analysis.

Note: Maximum likelihood estimation. Standardized estimates for associations and residuals (e). N=476.

Supplementary Table 23. Discriminant validity of the knowledge section

| **Secondary items** | **A3** | **A2** | **A1** |
| --- | --- | --- | --- |
| A3 | 0.854 |  |  |
| A2 | 0.735** | 0.821 |  |
| A1 | 0.622** | 0.522** | 0.821 |
| AVE | 0.729 | 0.675 | 0.674 |

Note: **denotes *P* < 0.001.

Supplementary Table 24. Discriminant validity of the section of practical skills

| **Secondary items** | **B4** | **B3** | **B2** | **B1** |
| --- | --- | --- | --- | --- |
| B4 | 0.882 |  |  |  |
| B3 | 0.544** | 0.875 |  |  |
| B2 | 0.581** | 0.875** | 0.856 |  |
| B1 | 0.458** | 0.706** | 0.731** | 0.754 |
| AVE | 0.778 | 0.766 | 0.733 | 0.569 |

Note: **denotes *P* < 0.001.

Supplementary Table 25. Discriminant validity of the leadership section

| **Secondary items** | **C5** | **C4** | **C3** | **C2** | **C1** |
| --- | --- | --- | --- | --- | --- |
| C5 | 0.908 |  |  |  |  |
| C4 | 0.760** | 0.822 |  |  |  |
| C3 | 0.730** | 0.742** | 0.901 |  |  |
| C2 | 0.799** | 0.821** | 0.779** | 0.857 |  |
| C1 | 0.819** | 0.789** | 0.829** | 0.834** | 0.895 |
| AVE | 0.825 | 0.675 | 0.811 | 0.734 | 0.801 |

Note: **denotes *P*< 0.001.

Supplementary Table 26. Discriminant validity of the section of personal quality

| Secondary items | D2 | D1 |
| --- | --- | --- |
| D2 | 0.751 |  |
| D1 | 0.653** | 0.885 |
| AVE | 0.564 | 0.783 |

Note: **denotes *P* < 0.001.

Supplementary Table 27. Model fit of four primary sections

| **Primary item** | χ²/df | GFI | CFI | NFI | TLI | SRMR | RMSEA |
| --- | --- | --- | --- | --- | --- | --- | --- |
| A Knowledge | 3.844 | 0.896 | 0.956 | 0.942 | 0.947 | 0.030 | 0.077 |
| B Practical skills | 2.258 | 0.930 | 0.976 | 0.959 | 0.972 | 0.026 | 0.051 |
| C Leadership | 2.235 | 0.922 | 0.978 | 0.961 | 0.974 | 0.018 | 0.051 |
| D Personal quality | 6.446 | 0.923 | 0.943 | 0.934 | 0.922 | 0.023 | 0.107 |
| **Criteria** | **<3** | **>0.9** | **>0.9** | **>0.9** | **>0.9** | **<0.05** | **<0.08** |

Note: GFI, goodness of fit index; CFI, comparative fit index; NFI, normed fit index; TLI, Tucker-Lewis index; SRMR, standardized root mean square residual; RMSEA, root mean square error of approximation.

Supplementary Table 28. Judgment matrix and consistency test of the primary items

| **Primary items** | **A** | **B** | **C** | **D** | **Weight** |
| --- | --- | --- | --- | --- | --- |
| A | 1 | 2 | 2 | 3 | 0.4063 |
| B | 1/2 | 1 | 2 | 3 | 0.2875 |
| C | 1/2 | 1/2 | 1 | 3 | 0.2081 |
| D | 1/3 | 1/3 | 1/3 | 1 | 0.0981 |
| Consistency ratio | 0.0456 | | | | |

Supplementary Table 29. Judgment matrix and consistency test of the knowledge section

| **A** | **A1** | **A2** | **A3** | **Weight** |
| --- | --- | --- | --- | --- |
| A1 | 1 | 2 | 2 | 0.4905 |
| A2 | 1/2 | 1 | 2 | 0.3119 |
| A3 | 1/2 | 1/2 | 1 | 0.1976 |
| Consistency ratio | 0.0517 | | | |

Supplementary Table 30. Judgment matrix and consistency test of the section of practical skills

| **B** | **B1** | **B2** | **B3** | **B4** | **Weight** |
| --- | --- | --- | --- | --- | --- |
| B1 | 1 | 1/2 | 1/2 | 4 | 0.2134 |
| B2 | 2 | 1 | 1/2 | 4 | 0.2944 |
| B3 | 2 | 2 | 1 | 4 | 0.4166 |
| B4 | 1/4 | 1/4 | 1/4 | 1 | 0.0756 |
| Consistency ratio | 0.0457 | | | | |

Supplementary Table 31. Judgment matrix and consistency test of the leadership section

| **C** | **C1** | **C2** | **C3** | **C4** | **C5** | **Weight** |
| --- | --- | --- | --- | --- | --- | --- |
| C1 | 1 | 1 | 2 | 1/2 | 2 | 0.2086 |
| C2 | 1 | 1 | 2 | 1/2 | 2 | 0.2086 |
| C3 | 1/2 | 1/2 | 1 | 1/2 | 2 | 0.1420 |
| C4 | 2 | 2 | 2 | 1 | 3 | 0.3439 |
| C5 | 1/2 | 1/2 | 1/2 | 1/3 | 1 | 0.0969 |
| Consistency ratio | 0.0196 | | | | | |

Supplementary Table 32. Judgment matrix and consistency test of the section of personal quality

| D | D1 | D2 | Weight |
| --- | --- | --- | --- |
| D1 | 1 | 1/4 | 0.2 |
| D2 | 4 | 1 | 0.8 |
| Consistency ratio | 0 | | |

Supplementary Table 33. Judgment matrix and consistency test of A1 Knowledge of infectious diseases

| A1 | A1_1 | A1_2 | A1_3 | A1_4 | Weight |
| --- | --- | --- | --- | --- | --- |
| A1_1 | 1 | 2 | 3 | 2 | 0.4129 |
| A1_2 | 1/2 | 1 | 3 | 1 | 0.2440 |
| A1_3 | 1/3 | 1/3 | 1 | 1/3 | 0.0992 |
| A1_4 | 1/2 | 1 | 3 | 1 | 0.244 |
| Consistency ratio | 0.0228 | | | | |

Supplementary Table 34. Judgment matrix and consistency test of A2 Knowledge of public health emergency management

| A2 | A2_1 | A2_2 | A2_3 | A2_4 | A2_5 | A2_6 | Weight |
| --- | --- | --- | --- | --- | --- | --- | --- |
| A2_1 | 1 | 3 | 3 | 2 | 1 | 1/2 | 0.2035 |
| A2_2 | 1/3 | 1 | 1/2 | 1/2 | 1/3 | 1/4 | 0.0628 |
| A2_3 | 1/3 | 2 | 1 | 1/2 | 1/3 | 1/3 | 0.0854 |
| A2_4 | 1/2 | 2 | 2 | 1 | 1/2 | 1/3 | 0.1187 |
| A2_5 | 1 | 3 | 3 | 2 | 1 | 1/2 | 0.2035 |
| A2_6 | 2 | 4 | 3 | 3 | 2 | 1 | 0.3262 |
| Consistency ratio | 0.0182 | | | | | | |

Supplementary Table 35. Judgment matrix and consistency test of A3 Laws, plans and mechanisms for responding to public health emergencies

| A3 | A3_1 | A3_2 | A3_3 | A3_4 | A3_5 | Weight |
| --- | --- | --- | --- | --- | --- | --- |
| A3_1 | 1 | 2 | 2 | 2 | 2 | 0.3244 |
| A3_2 | 1/2 | 1 | 2 | 1 | 2 | 0.2111 |
| A3_3 | 1/2 | 1/2 | 1 | 1/2 | 2 | 0.1444 |
| A3_4 | 1/2 | 1 | 2 | 1 | 2 | 0.2111 |
| A3_5 | 1/2 | 1/2 | 1/2 | 1/2 | 1 | 0.1089 |
| Consistency ratio | 0.0306 | | | | | |

Supplementary Table 36. Judgment matrix and consistency test of B1 Infectious diseases prevention and emergency preparedness

| B1 | B1_1 | B1_2 | B1_3 | B1_4 | B1_5 | Weight |
| --- | --- | --- | --- | --- | --- | --- |
| B1_1 | 1 | 2 | 1/2 | 1/2 | 1/2 | 0.142 |
| B1_2 | 1/2 | 1 | 1/3 | 1/2 | 1/2 | 0.0969 |
| B1_3 | 2 | 3 | 1 | 2 | 2 | 0.3439 |
| B1_4 | 2 | 2 | 1/2 | 1 | 1 | 0.2086 |
| B1_5 | 2 | 2 | 1/2 | 1 | 1 | 0.2086 |
| Consistency ratio | 0.0196 | | | | | |

Supplementary Table 37. Judgment matrix and consistency test of B2 Infectious diseases surveillance and early warning

| B2 | B2_1 | B2_2 | B2_3 | B2_4 | B2_5 | B2_6 | Weight |
| --- | --- | --- | --- | --- | --- | --- | --- |
| B2_1 | 1 | 1/2 | 2 | 2 | 1/2 | 1 | 0.1547 |
| B2_2 | 2 | 1 | 2 | 2 | 1 | 2 | 0.2440 |
| B2_3 | 1/2 | 1/2 | 1 | 1/2 | 1/2 | 1/2 | 0.0894 |
| B2_4 | 1/2 | 1/2 | 2 | 1 | 1/2 | 1/2 | 0.1133 |
| B2_5 | 2 | 1 | 2 | 2 | 1 | 2 | 0.2440 |
| B2_6 | 1 | 1/2 | 2 | 2 | 1/2 | 1 | 0.1547 |
| Consistency ratio | 0.026 | | | | | | |

Supplementary Table 38. Judgment matrix and consistency test of B3 Public health response to infectious diseases

| B3 | B3_1 | B3_2 | B3_3 | B3_4 | B3_5 | Weight |
| --- | --- | --- | --- | --- | --- | --- |
| B3_1 | 1 | 1/2 | 2 | 1 | 1/2 | 0.1591 |
| B3_2 | 2 | 1 | 2 | 2 | 1/2 | 0.2428 |
| B3_3 | 1/2 | 1/2 | 1 | 1/2 | 1/3 | 0.0965 |
| B3_4 | 1 | 1/2 | 2 | 1 | 1/2 | 0.1591 |
| B3_5 | 2 | 2 | 3 | 2 | 1 | 0.3426 |
| Consistency ratio | 0.0195 | | | | | |

Supplementary Table 39. Judgment matrix and consistency test of B4 Scientific research ability

| B4 | B4_1 | B4_2 | B4_3 | Weight |
| --- | --- | --- | --- | --- |
| B4_1 | 1 | 2 | 1/2 | 0.3119 |
| B4_2 | 1/2 | 1 | 1/2 | 0.1976 |
| B4_3 | 2 | 2 | 1 | 0.4905 |
| Consistency ratio | 0.0517 | | | |

Supplementary Table 40. Judgment matrix and consistency test of C1 Leadership fundamentals

| C1 | C1_1 | C1_2 | C1_3 | C1_4 | Weight |
| --- | --- | --- | --- | --- | --- |
| C1_1 | 1 | 3 | 2 | 2 | 0.4168 |
| C1_2 | 1/3 | 1 | 1/2 | 1/2 | 0.1209 |
| C1_3 | 1/2 | 2 | 1 | 1/2 | 0.1928 |
| C1_4 | 1/2 | 2 | 2 | 1 | 0.2695 |
| Consistency ratio | 0.0267 | | | | |

Supplementary Table 41. Judgment matrix and consistency test of C2 Decision-making ability

| C2 | C2_1 | C2_2 | C2_3 | C2_4 | C2_5 | Weight |
| --- | --- | --- | --- | --- | --- | --- |
| C2_1 | 1 | 1/2 | 1/2 | 1/3 | 1/2 | 0.0969 |
| C2_2 | 2 | 1 | 2 | 1/2 | 1 | 0.2086 |
| C2_3 | 2 | 1/2 | 1 | 1/2 | 1/2 | 0.1420 |
| C2_4 | 3 | 2 | 2 | 1 | 2 | 0.3439 |
| C2_5 | 2 | 1 | 2 | 1/2 | 1 | 0.2086 |
|  | 0.0196 | | | | | |

Supplementary Table 42. Judgment matrix and consistency test of C3 Team mobilization ability

| C3 | C3_1 | C3_2 | C3_3 | Weight |
| --- | --- | --- | --- | --- |
| C3_1 | 1 | 1/2 | 2 | 0.3119 |
| C3_2 | 2 | 1 | 2 | 0.4905 |
| C3_3 | 1/2 | 1/2 | 1 | 0.1976 |
| Consistency ratio | 0.0517 | | | |

Supplementary Table 43. Judgment matrix and consistency test of C4 Self-regulation and communication abilities

| C4 | C4_1 | C4_2 | C4_3 | C4_4 | C4_5 | C4_6 | Weight |
| --- | --- | --- | --- | --- | --- | --- | --- |
| C4_1 | 1 | 2 | 2 | 3 | 2 | 2 | 0.2887 |
| C4_2 | 1/2 | 1 | 1 | 2 | 2 | 1/2 | 0.1522 |
| C4_3 | 1/2 | 1 | 1 | 2 | 2 | 1/2 | 0.1522 |
| C4_4 | 1/3 | 1/2 | 1/2 | 1 | 1/2 | 1/2 | 0.0798 |
| C4_5 | 1/2 | 1/2 | 1/2 | 2 | 1 | 1/2 | 0.1108 |
| C4_6 | 1/2 | 2 | 2 | 2 | 2 | 1 | 0.2164 |
| Consistency ratio | 0.0273 | | | | | | |

Supplementary Table 44. Judgment matrix and consistency test of C5 Team learning and development

| C5 | C5_1 | C5_2 | C5_3 | Weight |
| --- | --- | --- | --- | --- |
| C5_1 | 1 | 2 | 1/2 | 0.3119 |
| C5_2 | 1/2 | 1 | 1/2 | 0.1976 |
| C5_3 | 2 | 2 | 1 | 0.4905 |
| Consistency ratio | 0.0517 | | | |

Supplementary Table 45. Judgment matrix and consistency test of D1 Professional qualifications

| D1 | D1_1 | D1_2 | Weight |
| --- | --- | --- | --- |
| D1_1 | 1 | 1/3 | 0.25 |
| D1_2 | 3 | 1 | 0.75 |
| Consistency ratio | 0 | | |

Supplementary Table 46. Judgment matrix and consistency test of D2 Professional quality

| D2 | D2_1 | D2_2 | D2_3 | D2_4 | D2_5 | D2_6 | D2_7 | Weight |
| --- | --- | --- | --- | --- | --- | --- | --- | --- |
| D2_1 | 1 | 1/2 | 2 | 1/2 | 1/2 | 1/2 | 2 | 0.1119 |
| D2_2 | 2 | 1 | 2 | 2 | 1 | 2 | 2 | 0.2151 |
| D2_3 | 1/2 | 1/2 | 1 | 1/2 | 1/2 | 1/2 | 2 | 0.0923 |
| D2_4 | 2 | 1/2 | 2 | 1 | 1/2 | 1 | 2 | 0.1452 |
| D2_5 | 2 | 1 | 2 | 2 | 1 | 2 | 2 | 0.2151 |
| D2_6 | 2 | 1/2 | 2 | 1 | 1/2 | 1 | 2 | 0.1452 |
| D2_7 | 1/2 | 1/2 | 1/2 | 1/2 | 1/2 | 1/2 | 1 | 0.0751 |
| Consistency ratio | 0.0308 | | | | | | | |

Supplementary Table 47. Final scale in English and Chinese

| **Primary items** | **Secondary items** | **Tertiary items** |
| --- | --- | --- |
|  |  |  |
|  |  |  |
| A Knowledge 专业知识 | A1 Knowledge of infectious diseases 传染病知识 | A1_1 Basic epidemiological knowledge of infectious diseases (judgment of infection source and transmission route, understanding of susceptible population, influencing factors of infectious disease epidemic, etc.) 传染病的流行病学基础知识（传染源和传播途径的判断、对易感人群的了解、传染病流行的影响因素等） |
|  |  | A1_2 Criteria for judging aggregates epidemics and outbreaks of infectious diseases (including nosocomial infections) and common prevention and control measures 传染病聚集性疫情及暴发疫情（包括医院感染）的判断标准与常用预防控制措施 |
|  |  | A1_3 Etiology knowledge and clinical knowledge of common infectious diseases 常见传染病的病原学知识和临床知识 |
|  |  | A1_4 Mastering knowledge and skills of personal protection 掌握传染病个人防护的知识和技能 |
|  | A2 Knowledge of public health emergency management 公共卫生应急管理知识 | A2_1 Fundamentals of public health emergency management 公共卫生应急管理与突发公共卫生事件的基础知识 |
|  |  | A2_2 Theories of health emergency management 卫生应急管理过程理论 |
|  |  | A2_3 Theories of crisis decision making 危机决策理论 |
|  |  | A2_4 Theories of risk assessment 风险评估理论 |
|  |  | A2_5 Theories of risk communication 风险沟通 |
|  |  | A2_6 Command, coordination and control of health emergency 卫生应急的指挥、协调与控制 |
|  | A3 Laws, plans and mechanisms for responding to public health emergencies 突发公共卫生事件应对的法律、预案和机制 | A3_1 The knowledge of relevant laws and regulations on infectious disease prevention and control and health emergency in China 对我国传染病防控和卫生应急相关法律法规的了解程度 |
|  |  | A3_2 The awareness of infectious disease prevention and control and health emergency plans at provincial and municipal levels 对所在省市层面的传染病防控和卫生应急相关预案的了解程度 |
|  |  | A3_3 Prevention and preparedness mechanism for public health emergencies in China 对我国突发公共卫生事件的预防准备机制的了解程度 |
|  |  | A3_4 Surveillance and early warning mechanism for public health emergencies in China 对我国突发公共卫生事件监测预警机制的了解程度 |
|  |  | A3_5 Response and rescue mechanism for public health emergencies in China 对我国突发公共卫生事件处置救援机制的了解程度 |
| B Practical skills 实践技能 | B1 Infectious diseases prevention and emergency preparedness 传染病预防与应急准备 | B1_1 Development of emergency plans 参与预案编制 |
|  |  | B1_2 Health popularization on infectious diseases and public health emergencies 开展对于传染病及卫生应急的健康科普 |
|  |  | B1_3 Receiving professional training  接受专业培训 |
|  |  | B1_4 Participating in emergency drills  参加应急演练 |
|  |  | B1_5 Emergency capacity assessment 能对本单位或本科室的应急能力进行评估 |
|  | B2 Infectious diseases surveillance and early warning 传染病监测与预警 | B2_1 Clarifying the content and process of infectious diseases surveillance 明确传染病监测的内容和流程 |
|  |  | B2_2 Ability to detect abnormal signals of public health emergencies 发现突发公共卫生事件异常信号的能力 |
|  |  | B2_3 Clarifying the reporting process for public health emergencies 明确突发公共卫生事件的报告流程 |
|  |  | B2_4 Determining the reliability of information sources on infectious diseases 能判定传染病信息来源的可靠性 |
|  |  | B2_5 Ability to extract key information from selected information sources 能从选择的信息来源中获取关键信息 |
|  |  | B2_6 Predicting occurrence and epidemic trends of infectious diseases based on surveillance information 能基于传染病监测信息，预测传染病的发生流行趋势 |
|  | B3 Public health response to infectious diseases 传染病的公共卫生应对 | B3_1 Clarifying on-site processing procedures for public health emergencies 明确突发公共卫生事件现场处理的流程 |
|  |  | B3_2 Mastering the principles of defining epidemic areas  掌握划定疫区的原则 |
|  |  | B3_3 Familiar with the methods of case management, specimen collection, and disinfection in the field environment 掌握病例处置、标本采集和现场环境消杀的方法 |
|  |  | B3_4 Ability to correctly carry out epidemiological investigations and write investigation reports 正确实施流行病学调查和流调报告撰写的能力 |
|  |  | B3_5 Analyzing the situation of public health incidents and proposing targeted prevention and control measures 分析突发事件的基本情况并提出针对性的预防控制措施 |
|  | B4 Scientific research ability 科学研究能力 | B4_1 Research design ability 具备研究设计能力 |
|  |  | B4_2 Ability to write research papers independently 能独立撰写科研论文或项目申请书 |
|  |  | B4_3 Understanding domestic and foreign status and trends in the profession 了解本专业内容国内外现状和趋势 |
| C Leadership 领导力 | C1 Leadership fundamentals 领导力基本素养 | C1_1 Proper allocation of tasks 具备任务合理分配能力 |
|  |  | C1_2 Ability to obtain resources needed for teamwork 能获取团队工作所需资源 |
|  |  | C1_3 Ability to allocate and dispatch resources needed for teamwork 能合理调度和分配团队工作所需资源 |
|  |  | C1_4 Supervising and adjusting project implementation 能对项目实施过程进行监督与调整 |
|  | C2 Decision-making ability 分析决策能力 | C2_1 Systematic understanding of current public health issues 对目前的公共卫生问题有系统的理解 |
|  |  | C2_2 Integrating different perspectives during decision making 做决策时能综合和整合不同观点 |
|  |  | C2_3 Clarifying problems to be solved and expected outcomes of decisions 明确待解决的问题和决策的预期结果 |
|  |  | C2_4 Ability to formulate alternative plans and select the optimal one 能拟定备选方案并选择最优方案 |
|  |  | C2_5 Ability to execute projects 能妥善执行项目 |
|  | C3 Team mobilization ability 团队动员能力 | C3_1 Creating an environment conducive to opinion exchange within the department 能在部门内营造利于意见交流的环境 |
|  |  | C3_2 Having characteristics to enable effective team leadership like integrity, enthusiasm, honesty, caring, trustworthiness, sense of responsibility, etc. 拥有让团队形成有效领导的特征，包括诚信、热情、诚实、关怀、信任、责任感等 |
|  |  | C3_3 Establishing effective team motivation models including listening, dialoguing, negotiating, rewarding, encouragement, inspiration, etc. 能建立有效的团队动员模式，包括倾听、对话、谈判、奖励、鼓励、激励等 |
|  | C4 Self-regulation and communication abilities 自我调节与沟通能力 | C4_1 Ability to communicate and coordinate with superiors, subordinates and partners 具备与上下级及合作伙伴有效沟通协调的能力 |
|  |  | C4_2 Use the media, where permitted by the organization, to regularly communicate relevant information about public health needs, goals, achievements, and major crises to target audiences 能在组织允许的情况下使用媒体定期与目标受众交流有关公共卫生需求、目标、成就以及重大危机的相关信息 |
|  |  | C4_3 Effectively applying negotiation skills in resolving disputes 能在调和争议时有效运用协商技巧 |
|  |  | C4_4 Understanding the impact of own behaviors or reactions on team members 了解自身行为或反应对团队成员的影响 |
|  |  | C4_5 Giving proper feedback to criticisms on own behaviors or performance by others 对于他人给自己行为或表现提出的批评给予恰当的反馈 |
|  |  | C4_6 Pressure toughness and ability to deal with complex problems 具备良好的抗压韧性及处理复杂问题的能力 |
|  | C5 Team learning and development 团队学习与发展 | C5_1 Identifying opportunities for team growth, innovation, reform and development 能识别团队成长、创新、变革和发展的机会 |
|  |  | C5_2 Creating opportunities for teams to learn and improve together 能创造团队共同学习和提升实力的机会 |
|  |  | C5_3 Helping members clarify thinking and turn ideas into feasible plans 能帮助成员理清思路，并将想法变成可行的计划 |
| D Personal quality 个人素养 | D1 Professional qualifications 从业资质 | D1_1 Having the level of education or professional training to meet the requirements of the job 具有满足工作要求的学历水平或通过了专业培训 |
|  |  | D1_2 The major was related to infectious disease prevention and control or health emergency 所学专业与传染病防控或卫生应急相关 |
|  | D2 Professional quality 职业素养 | D2_1 Physical fitness 具备良好的身体素质 |
|  |  | D2_2 Psychological quality 具备良好的心理素质 |
|  |  | D2_3 Political literacy 具备良好的政治素养 |
|  |  | D2_4 Abiding by the work standard and assuming the work responsibility 能自觉遵守工作规范、承担工作责任 |
|  |  | D2_5 Individual willingness and ability to learn 具备较强个人学习意愿和能力 |
|  |  | D2_6 Understanding my own work role and carry out appropriate work 清楚个人工作角色并开展适宜的工作 |
|  |  | D2_7 Training and guiding ability (guiding students or subordinates) 具备培训指导能力（指导学生或下级） |
